# Evolving a plant-beneficial bacterium in soil vs. nutrient-rich liquid culture has contrasting effects on in-soil fitness

**DOI:** 10.1128/aem.02085-24

**Published:** 2025-03-11

**Authors:** Laura M. Kaminsky, Liana Burghardt, Terrence H. Bell

**Affiliations:** 1 Boyce Thompson Institute 53401 , Ithaca, New York, USA; 2 Department of Plant Pathology and Environmental Microbiology, The Pennsylvania State University 171650 , University Park, Pennsylvania, USA; 3 Intercollege Graduate Degree Program in Ecology, The Pennsylvania State University, University Park, Pennsylvania, USA; 4 Department of Plant Science, The Pennsylvania State University 311373 , University Park, Pennsylvania, USA; 5 Department of Physical and Environmental Sciences, University of Toronto Scarborough 686144 , Toronto, Ontario, Canada; University of Tennessee at Knoxville, Knoxville, Tennessee, USA

**Keywords:** experimental evolution, bacterial adaptation, genetic pleiotropy, sporulation, plant growth-promoting bacteria

## Abstract

Inoculation of plant-beneficial microbes into agricultural soils can improve crop growth, but such outcomes depend on microbial survival. Here, we assessed how exposure to prior environmental conditions impacts microbial in-soil fitness, particularly focusing on incubation in liquid culture as an unavoidable phase of inoculant production and on pre-incubation in target soils as a potential method to improve performance. We conducted experimental evolution on a phosphorus-solubilizing bacterial species, *Priestia megaterium*, in (i) soil only, (ii) liquid media only, and (iii) soil followed by liquid media, using population metagenomic sequencing to track mutations over time. Several typical *in vitro* evolutionary phenomena were observed in liquid media-incubated populations, including clonal interference, genetic hitchhiking, and mutation parallelism between replicate populations, particularly in the sporulation transcription factor *spo0A*. Liquid media-incubated populations also developed a clear fitness reduction in soil compared to the ancestral isolate. However, soil-incubated populations grew slowly, experienced far fewer generations despite longer absolute time, and accumulated minimal mutational changes. Correspondingly, soil-incubated populations did not display improved survival compared to the ancestral isolate in their target soils, though there did appear to be minor fitness reductions in unfamiliar soil. This work demonstrates that adaptation to liquid media and/or a native soil can impact bacterial fitness in new soil and that bacterial evolution in more complex real-world habitats does not closely resemble bacterial evolution in liquid media.

**IMPORTANCE:**

Innovative solutions are needed to address emerging challenges in agriculture while reducing its environmental footprint. Management of soil microbiomes could contribute to this effort, as plant growth-promoting microorganisms provide key ecosystem services that support crops. Yet, inoculating beneficial microbes into farm soils yields unreliable results. We require a greater knowledge of the ecology of these taxa to improve their functioning in sustainable agroecosystems. In this report, we demonstrate that exposure to laboratory media and lingering adaptation to another soil can negatively impact the in-soil survival of a phosphorus-solubilizing bacterial species. We go further to highlight the underlying mutations that give rise to these patterns. These insights can be leveraged to improve our understanding of how soil-dwelling beneficial microorganisms adapt to different evolutionary pressures.

## INTRODUCTION

The presence of plant-beneficial microbes in agricultural soils can substantially improve crop growth and health outcomes through activities such as nutrient solubilization and pathogen suppression (1, 2). While microbe-driven plant growth promotion is often observed in the laboratory, greenhouse, and initial field trials, transferring these outcomes to the agricultural sector remains elusive. In particular, the performance of agricultural microbial inoculants is generally unreliable and unpredictable across broad ranges of soils (3–7). One factor in this unreliability is that inoculated microbes may simply not survive in the target soil (8), constraining their ability to develop an active growth-promoting population around crop roots. Methods that improve inoculant adaptation to and survival in particular soils could therefore help to counteract these issues and make microbial inoculants a more useful applied tool.

To this end, lab-based experimental evolution studies show that microorganisms can rapidly adapt to new environments and stressors (9–11). Simultaneously monitoring genomic and fitness changes in evolving populations can also yield fundamental insight into microbial evolutionary dynamics and the genetic basis for adaptation (12, 13). However, evolutionary patterns and adaptive outcomes observed in homogeneous media may not extend to the complex real-world habitats occupied by microbes (13–16). Soil habitats, for instance, are remarkably heterogeneous (17), with pH, nutrient content, moisture, oxygen availability, and other properties varying at the mm scale and across time (18, 19). These complex selective pressures are less likely to produce the simple sweeps of advantageous mutations observed in classic experimental evolution studies in liquid media (20). Indeed, experiments incorporating even slightly more spatial heterogeneity (e.g., static liquid media or agar with spatial abiotic gradients) tend to show more genetic and phenotypic diversity within the evolving population (21, 22). Still, soil-evolved isolates have been shown to outperform their ancestral isolate in soil fitness tests (23–25), pointing to soil-based experimental evolution as a possible means to improve microbial inoculant performance. There are some studies that have investigated microbial evolution in complex soil environments (23, 24, 26–29), but these remain far outnumbered by studies conducted in laboratory media. Further work is needed to explore how microbial taxa of interest respond and adapt to real-world soil environments.

Another consideration in studying and deploying agriculturally beneficial microbial isolates in the field is that they must pass through two very distinct environments: (i) simple, artificial media during laboratory handling and large-scale cell production in bio-fermenters (30–32) and (ii) any number of target soils different from the isolate’s soil environment of origin. The existence of such starkly different stages in the overall pipeline of inoculant capture, production, and deployment implies inherent trade-offs that complicate microbial inoculant success (33). For instance, bacteria rely on a wide set of tools to survive and grow in heterogeneous soil environments (34), but such bet-hedging strategies are often selected against in homogeneous, constant media environments, especially if they are energetically costly (35). Meanwhile, adaptation to one soil can compromise an isolate’s fitness in a different soil (25) if the traits that were neutral or beneficial in the face of certain soil abiotic properties and microbial residents are maladaptive in different soil contexts. More work is needed to untangle how these prior evolutionary signatures can result in antagonistic pleiotropy in novel soil environments. Specifically, we ask the following questions:

1. To what degree does passage in nutrient-rich lab media impact the survival of a microbial isolate in soil environments? Do we observe a reduction in microbial survival in the soil as a result of evolution in media, and could thus contribute to unreliable inoculant success?
2. Can pre-exposure to at least the abiotic conditions of a target soil improve the survival of a microbial isolate in that soil?
3. If there are fitness changes in post-inoculation soil survival in media- or soil-evolved populations, what are some of the genetic changes occurring in those populations that may be contributing?

To address these questions, we conducted experimental evolution on a plant-beneficial bacterium, *Priestia megaterium—* formerly *Bacillus megaterium* (36). *P. megaterium* acts as a free-living phosphorus solubilizer (37, 38), liberating phosphate from organic matter and insoluble complexes into the soil solution where it can be taken up by plant roots (39–41). Even though *P. megaterium* is often already present in agricultural soils (8, 42), it is a common species included in commercial microbial inoculants (43) because it has clear plant growth-promoting abilities, is easy to grow in the lab, and forms dormant endospores conducive to longer shelf stability (44). Yet, *P. megaterium* also displays variable survival upon inoculation into different soils, which vary in pH, organic matter levels, and nutrient content (8), tracking with theoretical frameworks suggesting that selective forces in the surrounding environment contribute to determining where certain microbes can survive (45). This makes *P. megaterium* a useful target organism for applied evolutionary strategies.

**Fig 1:**
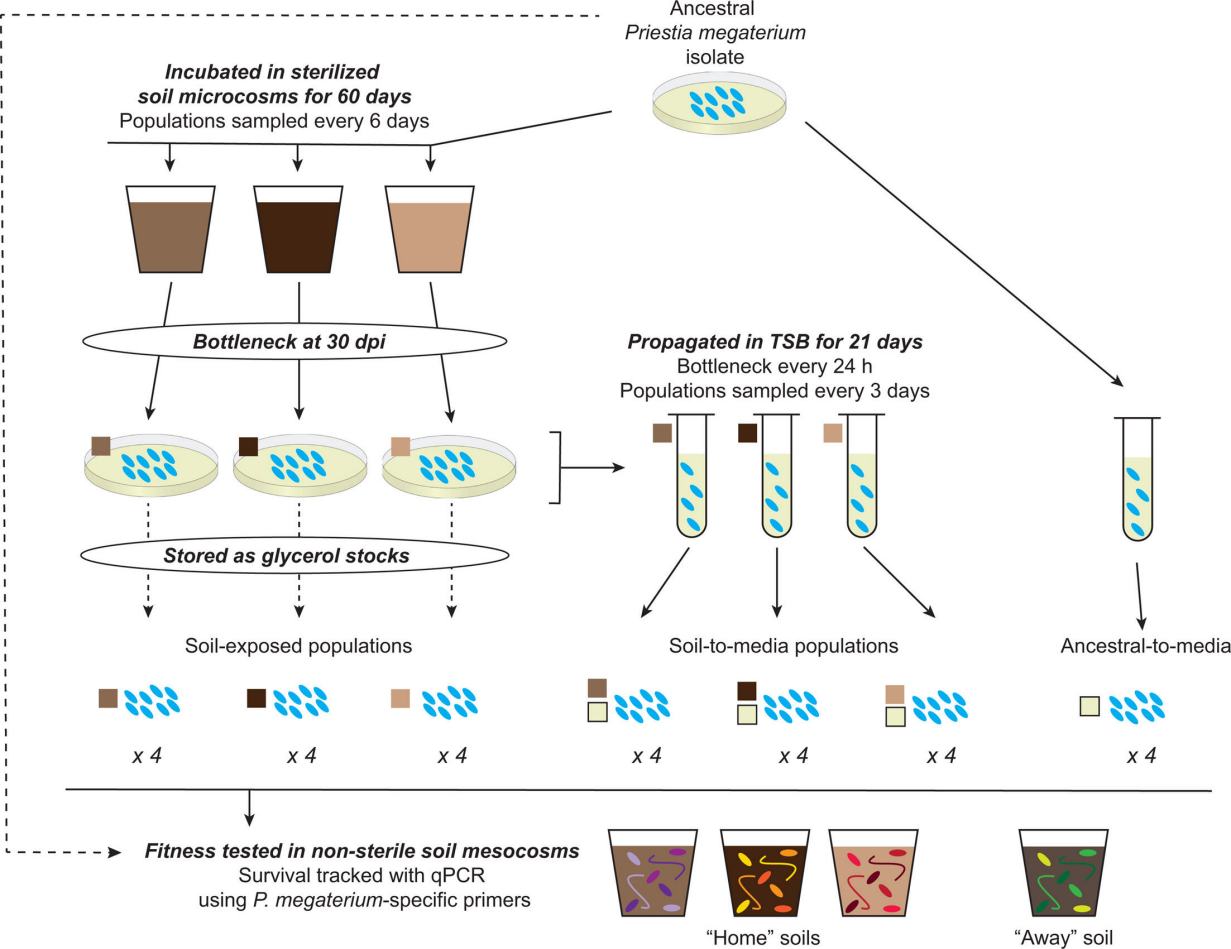
Experimental design and overview.

We used three different experimental evolution approaches, illustrated in Fig. 1. Approach 1 involved passaging *P. megaterium* populations in tryptic soy broth (TSB) for 21 days (ancestral-to-media populations). This allowed us to assess how a soil-dwelling microbe responds to the selective pressures inherent in extended laboratory handling and acted as a control for approaches 2 and 3. The 21 days for this phase was meant to approximate the evolutionary time an isolate might experience during laboratory domestication (31), isolate screening and testing, and cell production for product formulation (46). Approach 2 involved incubation of *P. megaterium* populations in three separate, sterilized soils with varying abiotic properties (Table S1) for 60 days (soil-exposed populations). This allowed us to trial soil-based experimental evolution as a potential method to increase inoculant fitness, decipher selective pressures in soil environments, and compare liquid media- vs. soil-based evolutionary patterns. We chose 60 days for this phase because, in a prior experiment, we found that a population of *P. megaterium* incubated in similar soil microcosms under the same conditions had accumulated a notable number of mutations in just 30 days (Kaminsky et al. submitted), so we expected similar or greater evolutionary progress. In addition, other experimental evolution studies in soil ran for similar timeframes (26, 28). Finally, approach 3 involved incubating the soil-exposed populations in TSB for 21 days (soil-to-media populations). This allowed us to assess the stability of any soil-acquired evolutionary changes even after evolution in media. We tracked mutations within the evolving populations via shotgun metagenomic sequencing and then tested for antagonistic pleiotropy effects by tracking microbial survival following inoculation into non-sterile microbially diverse soils.

We hypothesized that incubation in a target soil would result in (i) a fitness improvement in that same soil compared to the ancestral isolate, with potential fitness trade-offs in other soils and (ii) the accumulation of mutations corresponding to different abiotic stressors present in each soil type. We further predicted that incubation of the ancestral isolate and the soil-exposed populations in liquid media would result in (iii) a shift in dominant mutations, with novel media-specific mutations increasing in frequency and soil-associated mutations decreasing in frequency; and (iv) a decrease in fitness in soil environments compared to the ancestral isolate, as mutations adaptive or neutral in nutrient-rich media are often deleterious in more ecologically relevant conditions. By directly comparing the evolution of one ancestral isolate in soil vs. liquid culture, we reveal patterns of microbial adaptation to the two contrasting environments in which inoculant microorganisms must survive and function.

## MATERIALS AND METHODS

### Ancestral isolate

Our ancestral isolate was derived from the commercial microbial product MegaPhos (Blacksmith BioScience Inc., Spring, TX, USA), advertised as a phosphorus-solubilizing agent containing only *Priestia megaterium* and labeled for broad use on crop plants as a soil drench, seed treatment, or hydroponic additive. MegaPhos powder was suspended in sterile water and plated on tryptic soy agar (TSA) to obtain *P. megaterium* colonies (confirmed with whole-genome sequencing, see details below). We note that information on the original source of the ancestral isolate was unavailable from the company, nor was the number of generations spent in a laboratory setting before distribution is known. We also made no further genetic changes to the ancestral isolate (e.g., tagging with a fluorescent or antibiotic marker) because we wished to avoid unintended fitness consequences of such changes and because we were interested from an applied standpoint in working with the commercial isolate as a farmer would receive it. The ancestral isolate was grown overnight in TSB, a portion of which was stored as glycerol stock at −80°C.

### Incubation in sterilized soil microcosms

To obtain material for our soil microcosms, we collected field soils in June 2021 from three locations: (i) a monoculture corn field in the Penn State Arboretum (AC); (ii) a fallow grassy field near the Penn State Community Garden (CG); and (iii) a monoculture soy field in the Russel E. Larson Agricultural Research Station (RS) (Table S1). The soils were then homogenized through a 2 mm sieve. To prepare each microcosm, 300 mL of sieved soil was placed in a lidded 565 mL round Microbox (SacO_2_ company, Deinze, Belgium), which contains a filter to allow gas exchange while excluding potential contaminating organisms. To remove resident soil microorganisms, the microcosms were autoclaved at 121°C for 60 minutes three times with a 24 h incubation at room temperature between rounds to kill any soil microorganisms exiting dormancy. Although gamma irradiation is often used for soil sterilization, both methods change soil abiotic properties, and we have found gamma irradiation less reliable at removing microbes and more costly than multiple rounds of autoclaving, which is supported by other studies (47, 48). In addition, subsequent microbial colonization is very similar across both methods (49). Our choice to remove resident microbes from these mesocosms despite our goal to improve inoculant fitness in non-sterile, microbial-diverse soils was driven by two factors: (i) We wanted to ensure that we could easily recapture our evolving *P. megaterium* populations, which was aided by the absence of other microbes and (ii) the composition of the soil microbiome in any given field can vary significantly across time (50), but the baseline abiotic properties of a soil are slower to change. We, therefore, reasoned that exposure to abiotic soil properties may provide a baseline selective environment that could influence inoculant fitness even in the face of fluctuating resident microbes. To test this hypothesis, we evolved *P. megaterium* in autoclaved soils but tested its fitness in non-sterile soils (see “Fitness test,” below).

Samples of both the pre-autoclaved soils (used later in fitness tests, see below) and the thrice-autoclaved version of each soil were submitted to the Penn State Ag Analytical Services Lab to characterize soil abiotic properties. Autoclaved soil AC had a near-neutral pH, while autoclaved soils CG and RS were more acidic and drier than soil AC, and there was considerable variation in nutrient content across the three soils (Table S1).

For each soil type, we included four replicate experimental microcosms plus one uninoculated negative control microcosm per soil type, for a total of 15 microcosms. Our inclusion of negative control microcosms allowed us to determine whether any contaminants survived autoclaving at high enough numbers to affect the focal *P. megaterium* population in the experimental microcosms and whether our sampling methods introduced contaminants.

To begin the soil incubation, ancestral *P. megaterium* was grown overnight in TSB, then spun down and re-suspended in water. A 10 mL aliquot of this suspension (~1.3 × 10^8^ CFUs in total) was added to each of the 300 mL soil microcosms, excepting the negative controls where 10 mL of autoclaved water was added instead. The microcosms were then placed in a humidity chamber and incubated at 20°C and 85% relative humidity for a total of 60 days, with one population bottleneck imposed at the halfway point (see “Population bottleneck at 30dpi” below).

### Population sampling in soil microcosms

Every 6 days throughout the soil incubation, the microcosms were sampled to check for contamination, and at four time points (6 dpi, 30 dpi, 36 dpi, and 60 dpi), we conducted metagenomic sequencing to track evolutionary changes over time. We opted to sequence DNA extracted from pools of 100 isolates collected from each microcosm (details below) rather than DNA extracted directly from soil samples because we have found that DNA yields from inoculated, thrice-autoclaved soil can be very low, especially at earlier time points. These low yields complicate library preparation and can make it difficult to obtain the deep coverage required for population-level mutation analysis without biased pre-amplification steps. In addition, other studies have used a similar isolate pooling approach to sample and compare evolved populations with the ancestral isolate but included far fewer isolates per population (between 5 and 20) for this characterization (24, 27, 29). While we likely missed some rare genetic variants in the population by focusing on 100-isolate pools, we were more focused on the mutations rising to detectable frequencies (e.g., greater than 1%) as these were more likely to explain any fitness change at the population level.

To obtain the 100-isolate pools, microcosms were vigorously shaken to homogenize the soil, sprayed with ethanol to disinfect the outside of the container, opened in a UV-sterilized biosafety cabinet to collect 2 mL soil samples with sterile single-use plastic scoops, then immediately closed and resealed with parafilm. A 50 mg subsample of soil was added to 5 mL autoclaved water and vortexed at maximum speed for 1 minute to create a soil slurry. Soil slurry dilutions were then plated on TSA and incubated for 2 days at room temperature. We then transferred 100 colonies with *P. megaterium* morphology onto gridded, 100 mm diameter TSA plates (one colony streaked diagonally per grid cell) and allowed the colonies to grow for 1 day. The gridded plates were then flooded with autoclaved distilled water, and the cells were suspended using sterile single-use cell spreaders. The cell suspension was then pipetted into 15 mL tubes and gently vortexed to homogenize. We used 1 mL of each cell suspension for metagenomic sequencing (see “Population re-sequencing throughout soil and media incubations,” below), and the remainder was stored as glycerol stock at −80°C.

We additionally used the soil slurry plates to document *P. megaterium* population growth with CFU counts, though we acknowledge that this approach did not distinguish between vegetative *P. megaterium* cells and spores. To estimate the number of generations experienced in a set amount of time from CFU counts, we used the following equation: Nt = N0 * e^0.6931x^, where N0 is the initial population size, Nt is the final population size, and x is the number of generations, here defined as the number of population doubling events that have occurred. We also checked each plate for contaminants with colony morphologies different from *P. megaterium*. Uninoculated control microcosms sometimes yielded such colonies on TSA, indicating that some microorganisms may have survived autoclaving or were introduced during microcosm sampling. However, the abundance of these contaminants was many orders of magnitude lower than the abundance of *P. megaterium* in the inoculated microcosms, and the inoculated microcosms almost exclusively yielded colonies with morphologies matching that of *P. megaterium*.

### Population bottleneck at 30dpi in soil microcosms

After 30 d of soil incubation, we imposed a population bottleneck on our *P. megaterium* populations by inoculating new, freshly autoclaved soil microcosms with the 30 dpi 100-isolate pools. This was to re-encourage active growth of *P. megaterium*, as CFU counts had stabilized (Fig. S1), suggesting that populations may have reached carrying capacity. To make new soil microcosms, we retrieved field soils that had been stored at 4°C and sieved and autoclaved them as before in new Microboxes. These were then inoculated with the appropriate 100-isolate mix from the 30 dpi sampling date. We standardized the number of cells added per microcosm to 1.3 × 10^8^ CFUs by calculating the appropriate cell suspension volume for each population from OD_600_ readings, adjusted to a total volume of 10 mL with sterile water. Negative control microcosms were inoculated with 10 mL of sterile water as before. The remaining 30 days of soil incubation were carried out as before, with microcosm sampling every 6 days.

### Incubation in liquid media

Following the 60 d soil incubation, we performed a 21 d incubation in TSB. This portion of the experiment included 16 experimental populations: the 12 endpoint populations from the soil incubation plus four replicates of the ancestral isolate inoculated into TSB. A 1 mL aliquot of glycerol stock of each population was thawed and centrifuged at 5,000 rpm for 3 min, then the supernatant was discarded, and the cell pellet was washed once with 1 mL of 0.9% NaCl. The cells were then re-suspended in 1 mL TSB and added to 9 mL TSB in a 50 mL sterile plastic tube to begin the TSB incubation. The cultures were incubated in a shaking incubator at 200 rpm and 30°C, alongside a negative control tube with 10 mL of uninoculated TSB. Every 24 h in a UV-sterilized biosafety cabinet, 0.1 mL of media was taken from the overnight cultures and inoculated into 9.9 mL of fresh TSB, a 1:100 population dilution. This continued for 21 days.

Every third day, aliquots of the overnight culture were (i) set aside for DNA extraction to track population-level genetic changes; (ii) diluted and plated on TSA for CFU counts and to check for contamination; and (iii) stored as glycerol stocks at −80°C. We did not observe contaminating colonies in our evolving populations nor growth in our negative controls, and we conclude that our cultures remained axenic throughout the 21 days of TSB incubation.

### Population re-sequencing throughout soil and media incubations

To extract DNA for metagenomic sequencing, we used the GenElute Bacterial Genomic DNA kit (Sigma-Aldrich Co., St. Louis, MO, USA) on 1 mL aliquots of the 100-isolate pools from the 6 dpi, 30 dpi, 36 dpi, and 60 dpi sampling points in the soil incubation and 1 mL aliquots of overnight culture from the 3 dpi, 6 dpi, 9 dpi, 12 dpi, 15 dpi, 18 dpi, and 21 dpi sampling points in the TSB incubation. We additionally extracted DNA from overnight cultures of the ancestral isolate and three randomly selected individual isolates from 60 dpi soil populations and 21 dpi TSB populations for whole-genome sequencing (WGS). In the case of the ancestral isolate, this was to provide a baseline reference genome for the experiment. In the case of the endpoint isolates, this was to determine whether any mutations detected in the overall population were co-localized within the same genome. DNA extracts were prepared for sequencing with the Illumina DNA PCR-free prep kit (Illumina, San Diego, CA, USA) and sequenced 2 × 150 bp on an Illumina NextSeq at the Cornell University Genomics Core Facility, at a goal depth of 60× for WGS samples and 1000× for metagenomic samples, comparable to that of other population-level mutation analysis studies (51).

### Bioinformatics

The read quality of all raw sequences was assessed with FastQC v0.11.9 (52) and low-quality bases were removed with Trimmomatic v0.39 using default parameters (53). The ancestral isolate genome was assembled *de novo* from trimmed reads with SPAdes v3.15.3 (54) using the “careful” option and k-mer lengths of 99 and 127. Average coverage was calculated using BWA v0.7.17 (55) and SAMtools v1.12 (56), and assembly quality (Table S2) was checked with QUAST v5.1.0rc1 (57). The assembled ancestral genome was annotated with Prokka v1.14.6 (58) using *Priestia megaterium* strain ATCC 14581 (https://www.ncbi.nlm.nih.gov/nuccore/CP069288) as a naming guide (59). This annotated genome from the ancestral isolate served as the reference genome against which to compare sequences from evolved populations and isolates.

To identify mutations in the evolving populations, we used breseq v0.36.1 (60), which maps each sequencing read to the reference genome and identifies and annotates genetic variation between the aligned reads and the reference genome. We used polymorphism mode for trimmed reads from metagenomic population samples, which provides an estimate of the population-level frequency of each mutation, and clonal mode for trimmed reads from single isolates. Mutations in three genes (recQ and two different hypothetical proteins) were discarded from all population mutation lists based on low-quality read alignment (60), their presence in over 80% of all samples in all soil types and TSB at all time points, and their high frequency (>25%) in the 6 dpi soil populations despite these populations experiencing no more than eight generations by that time, all of which suggested that these were consistent misalignments rather than true mutations. We considered all remaining mutations identified by breseq in our subsequent analyses rather than removing those with population-level frequencies below 10% (despite prior suggestions to use this conservative cut-off for whole population sequencing (15)) due to our deep sequencing depth. Moreover, in our single isolates, we frequently detected mutations present at <10% frequency in the corresponding 100-isolate pool, validating these as true genetic variants. All figures were generated in R v4.2.1.

We note that because we used short-read metagenomic sequencing, we focus our results on single-nucleotide polymorphisms and small insertions and deletions rather than larger structural changes. We also largely focus our analysis and discussion on non-synonymous mutations, as have other studies (28), as these have the most straightforward link to phenotypic change.

### Fitness test: survival in non-sterile soil microcosms

Finally, we tested the in-soil fitness of the ancestral isolate, soil-evolved populations, soil-to-media populations, and ancestral-to-media populations by tracking their population density following inoculation into non-sterile, microbially diverse soil microcosms out to 15 dpi using a set of quantitative PCR primers designed to target *P. megaterium* DNA within soil DNA extracts (8). We note that the gene region targeted by these primers never appeared in the mutation lists of any evolving population. Thus, these primers are expected to work equally well for the ancestral isolate and the evolved isolates.

While fitness changes following experimental evolution are more traditionally assessed with 1:1 competition of ancestral isolates vs. evolved isolates (12), we chose to assess each population independently. When two populations are presented with the same soil conditions and inoculated at the same concentration, and one survives for longer and at higher densities than the other, this is a strong indication that it is better adapted to those conditions. In addition, we focused on *P. megaterium* cell densities in soil over time rather than other metrics of microbial fitness. We felt this provided the most relevant data for answering the applied question of whether soil vs. media-based evolution impacts post-inoculation microbial survival. In addition, other fitness metrics, such as microbial growth rate, are difficult to measure in soil. In particular, current methodologies involving stable isotopes or radioactive element incorporation do not provide taxon-specific information (61), and growth rate estimates vary significantly with the methodology used (62). Moreover, the rate and pattern of microbial growth *in vitro* do not resemble what is observed *in situ* in soil environments, with generation times numbering in days to months for soil-dwelling microbes rather than minutes to hours for lab-grown microbes. Given this, we felt the simplest and best metric for assessing *P. megaterium* fitness was tracking survival over time.

Microcosms were prepared in the same manner as before from the AC, CG, and RS soils, except that they were not autoclaved before inoculation, 75 mL rather than 300 mL of soil was used per microcosm, and they were contained in 100 mm × 25 mm deep petri dishes rather than Microboxes. An additional soil was collected from the Cover Crop Cocktails site at the Russell E. Larson Agricultural Research Station (CCC) in June 2022 for use as an “away” soil in this assay, to determine if any observed fitness changes were soil-specific, leading to evolutionary trade-offs in a different soil environment. The CCC soil was prepared in the same manner as the others. We note that the unsterilized soils generally had much lower ammonium levels and slightly lower pH values than their autoclaved counterparts, but other properties remained in approximately the same range (Table S1).

Microcosm replication was as follows: (i) four uninoculated microcosms per soil type; (ii) four replicate microcosms inoculated with the ancestral isolate per soil type; and (iii) one replicate microcosm for each evolved population per soil type. Ancestral-to-media populations were tested in all four soils, while soil-evolved and soil-to-media populations were tested only in their “home” soil and the CCC “away” soil. Inoculum was prepared from overnight TSB culture of glycerol stocks of the ancestral isolate and evolved populations, and standardized to 1.3 × 10^8^ CFUs per microcosm added in 4 mL of sterilized water. For uninoculated controls, 4 mL of sterilized water was added instead. An equivalent ratio of cell suspension and water was added to 250 mg of the appropriate soil in a DNA extraction tube (NucleoSpin 96 Soil DNA extraction kit, Macherey-Nagel, Düren, Germany) for each population, and this was then immediately extracted and subjected to qPCR (details below). This served as an estimate of the 0 dpi *P. megaterium* population size in each microcosm.

At 2 dpi, 5 dpi, 8 dpi, 12 dpi, and 15 dpi, microcosms were non-destructively sampled to assess *P. megaterium* abundance. Microcosms were homogenized with vigorous shaking, then carefully opened in a UV-sterilized biosafety cabinet to collect 250 mg soil samples for DNA extraction using the NucleoSpin 96 Soil DNA extraction kit (Macherey-Nagel, Düren, Germany), with a final DNA elution in 200 µL 5 mM Tris/HCl pH 8.5 buffer. This DNA served as the template for qPCRs using the php primer set (php-F: 5′-AGCCCGCACGATATAAAGATGT-3′; php-R: 5′-GCAGCATGTCTTCCGCTTCA-3′) previously designed and vetted to quantify inoculated *Priestia megaterium* populations in soil samples (8). All qPCRs were performed on a Bio-Rad C1000 Touch Thermal Cycler and CFX96 Real-Time System machine. The 20 µL reactions were performed in technical triplicates and contained 10 µL of PCR Master Mix from the QuantiNova SYBR Green PCR kit (Qiagen), 1.4 µL of each primer at 10 µM (a final concentration of 700 nm in the reaction), 6.2 µL of sterile nuclease-free H_2_O, and 1 µL of DNA template. Cycling conditions were as follows: 95°C for 2 minutes, 40 cycles of 5 seconds melting at 95°C, and 10 seconds of combined annealing and extension at 60°C. Melting curve analysis consisted of 5 seconds at every 0.5°C interval from 65°C to 95°C. Quantification cycle (Cq) values and melting curve results were determined with the Bio-Rad CFX Maestro software using default settings. Cq values were converted to CFU g^−1^ dry soil using the calibration curves already generated for the AC, CG, and RS soils in Kaminsky and Bell (8). A calibration curve for the CCC soil was generated in the same manner for this study and used for Cq conversions for those soil samples.

### Statistical analyses

Linear models and ANOVA were used to test the effect of evolutionary history on *P. megaterium* population size in each soil at each time point in R v4.2.1, first confirming data normality and homogeneity of variance. Post hoc comparisons were made using Tukey’s HSD test (R package emmeans). Furthermore, independent two-sample t-tests were used to test the hypothesis that evolved populations had significantly different population sizes than the ancestral isolate at the final time point, 15 dpi.

## RESULTS

### Comparing evolutionary generations in soil vs. culture

By counting colony-forming units (CFUs), we estimate that the media-evolved populations experienced ~6.6 generations d^−1^. This equals 19.9 generations in the 3 days between each sampling point and 139.5 generations total. This daily growth rate is very similar to that observed in the classic long-term evolution of *Escherichia coli* (20). By contrast, population growth in soil was slow and mainly occurred after the initial inoculation and an experiment-imposed population bottleneck after 30 days (Fig. S1). In the first 6 days post-inoculation and post-bottleneck, we estimate that ~8.2 generations occurred on average, with some variance across soil types and time points. Therefore, it took ~4.8 days in soil to achieve the same growth as in 1 day in culture. After these periods of growth, generational estimates are difficult because population levels stabilized (Fig. S1), but the number of soil-based generations was likely substantially lower than the ~140 generations subsequently experienced in media. Because *P. megaterium* is an endospore-former (44), it is also possible that a portion of the soil-exposed populations formed spores, allowing them to avoid soil-based selective pressures rather than adapting to them. However, novel mutations were detected at every time point in the soil (Fig. S2A), indicating that these populations were not static and likely experienced turnover. Still, populations grew more quickly and consistently in culture despite the shorter time frame, and this difference in generation time is likely a key factor influencing our results.

### Impacts of experimental evolution on fitness in soil

**Fig 2:**
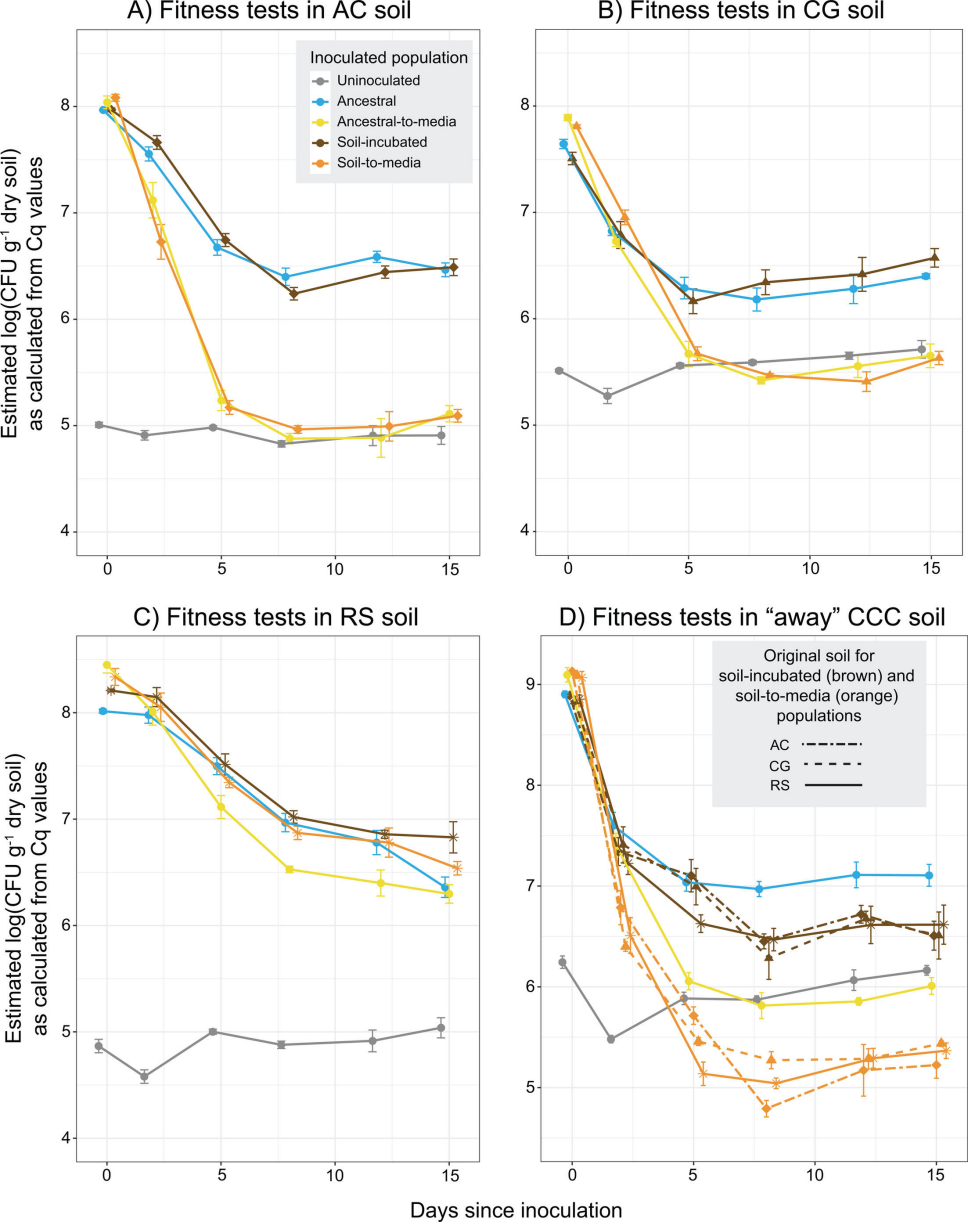
Population density of *P. megaterium* in the fitness test soil microcosms over time in four separate soils (panels A–D) , as determined by qPCR targeting *P. megaterium* within soil DNA extracts. Points represent the mean ± se for replicate microcosms. Each soil-evolved population and its TSB-evolved counterpart was tested both in its “home” soil and an “away” soil, CCC, while the ancestral isolate and ancestral-to-media populations were tested in all four soils. Results of ANOVA and Tukey HSD tests testing the effect of microcosm type on *P. megaterium* population density at each time point are included in Table S3. Soil type abbreviations: AC = Arboretum Cornfield, CG = Community Garden, RS = Russel E. Larson Agricultural Research Station, CCC = Cover Crop Cocktail site.

To determine the impact of isolate passaging in media vs. soil, we tested the fitness of the ancestral isolate, soil-evolved populations, soil-to-media populations, and ancestral-to-media populations by inoculating each into microbially diverse non-sterile soils and tracking their survival over time with qPCR targeting *P. megaterium* within soil DNA extracts (8). We note that *P. megaterium* was detected in our uninoculated controls (Fig. 2, grey lines) in line with findings that this bacterium is commonly found as part of the natural soil microbiome (8, 42). However, inoculation with our experimental *P. megaterium* consistently spiked the initial population size to many orders of magnitude above the uninoculated baseline. Each soil-evolved and soil-to-media population was tested in its “home” soil (AC, CG, or RS, Fig. 2Athrough C), in which it had been initially incubated, as well as a common “away” soil (CCC, Fig. 2D). The ancestral and ancestral-to-media *P. megaterium* were tested in all four soils.

**Fig 3:**
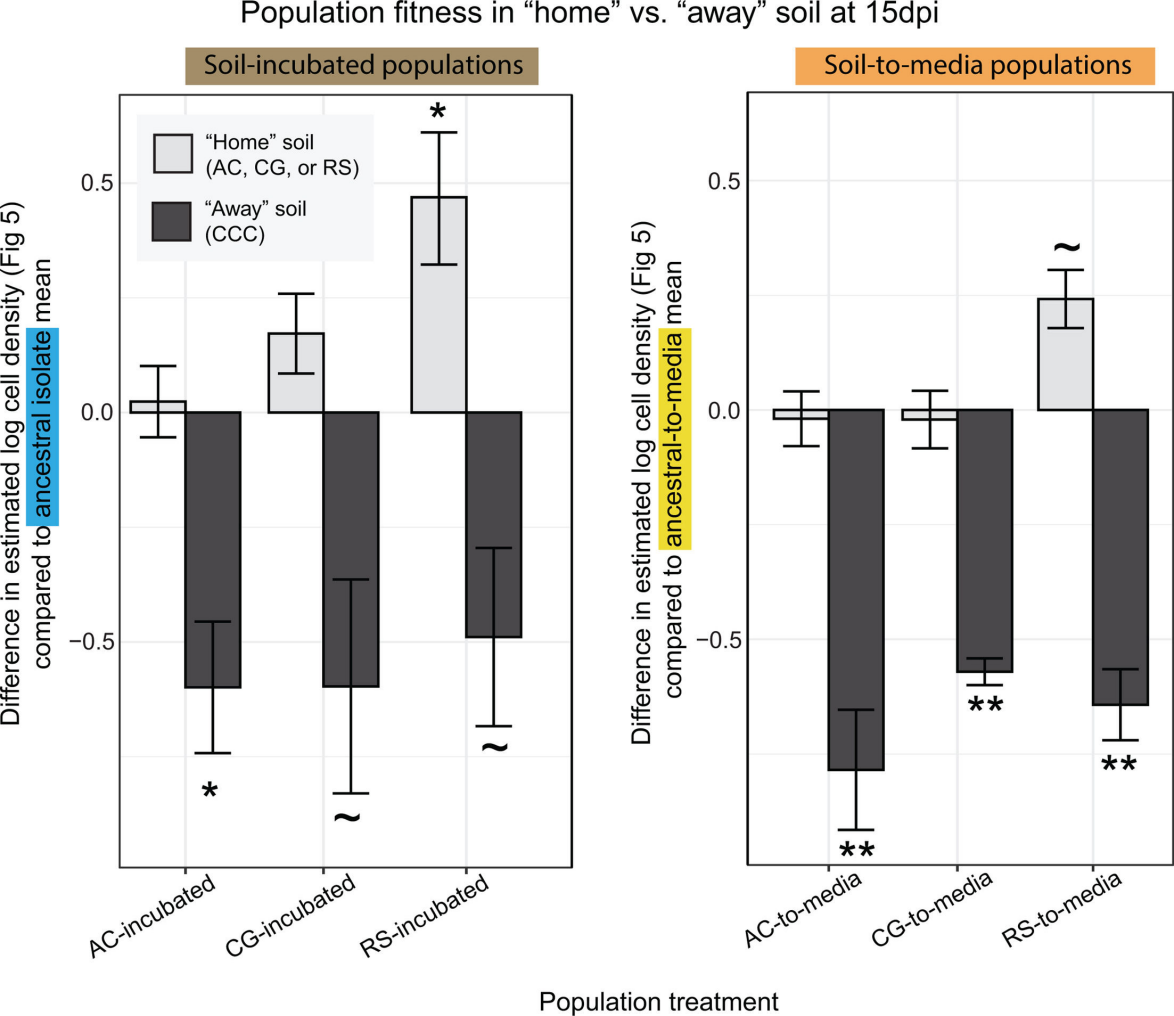
Fitness of evolved populations in “home” vs. “away” soils. Displayed are the mean ± SE difference in log cell density, as determined by qPCR targeting *P. megaterium,* between the ancestral isolate (or ancestral-to-media population) and the indicated evolved population at the final 15 dpi sampling point of the fitness test. Text highlight colors correspond with Fig. 2, while results in “home” vs. “away” soils are colored different shades of gray. Independent Welch two-sample t-tests were used to test the hypothesis that evolved populations had significantly different population sizes compared to the ancestral isolate (for soil-incubated populations) or ancestral-to-media populations (for soil-to-media populations) at the final time point (see Table S4 for test values). *P*-value symbols: ~<0.1; *<0.05; **<0.005.

In contrast to our predictions, none of the soil-evolved populations (brown lines) demonstrated a fitness advantage compared to the ancestral isolate (blue lines) in their “home” soil (Fig. 2A through C; Table S3), instead surviving across time at comparable levels. However, this outcome was reasonable given the potentially limited generational divergence of soil-incubated populations from the ancestral isolate. Yet, when the soil-evolved populations were inoculated into an unfamiliar “away” soil, there appeared to be a slight decrease in fitness compared to the ancestral isolate (Fig. 3). Even though this difference was generally not quite significant (Table S4 ), it could indicate a degree of adaptation specific to the soil of incubation and a corresponding trade-off in fitness in other soil environments. This fitness trade-off in the “away” soil appears to have been retained after incubation in media, as soil-to-media populations generally survived worse than ancestral-to-media populations (Fig. 3, Table S4).

By contrast, the liquid media-evolved populations (yellow and orange lines) demonstrated significantly decreased fitness compared to the ancestral isolate and soil-evolved populations in most soils, supporting our original prediction (Fig. 2, Table S3 ). The exception was in RS soil microcosms, where all populations survived at similar densities across time (Fig. 3C). This could be explained by a prior study in the same soils, which found the greatest inoculation success in RS soil (8), indicating that this environment may have been more compatible overall with our *P. megaterium*. Still, time spent in media generally resulted in a fitness disadvantage in soil environments.

### Mutation frequencies, retention across time, and parallelism

**Fig 4:**
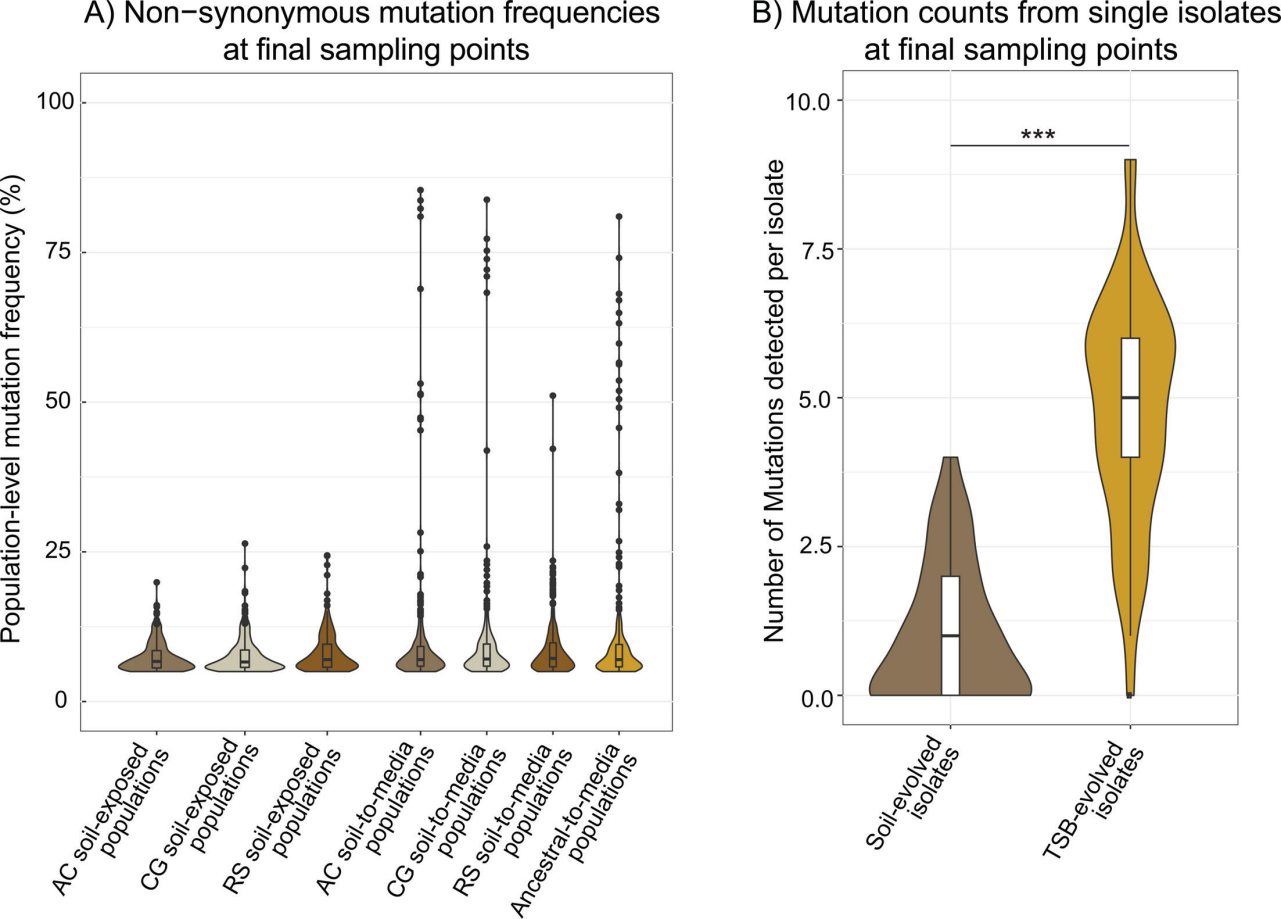
Mutation frequencies in soil vs. TSB-evolved populations. (**A**) Frequencies of all non-synonymous mutations were detected at the final sampling points (60 dpi for soil; 21 dpi for TSB). All mutations from each of the four replicate mesocosms of a given treatment are lumped and displayed together in one violin plot. (**B**) Distribution of the number of mutations detected in single isolates subjected to whole-genome sequencing after 60 days of soil incubation or 21 days of TSB incubation. *P*-value from Welch two-sample t-test between the two groups = 2.2e-16.

To investigate the potential genetic changes underlying these fitness results, we tracked mutations arising in the evolving populations during their passaging in soil and/or media. After 60 d, the highest frequency non-synonymous mutation in any soil-incubated population was only 26.4% (Fig. 4A). By contrast, 12 of the 16 TSB-incubated populations contained non-synonymous mutations at >50% frequency after 21 d. However, most mutations across the experiment remained below ~10% frequency (Fig. 4A) and were detected at only a single time point (Fig. S3A and B), indicating that they were unlikely to have been targets of positive selection. This reinforces the assertion that most novel mutations are likely neutral or deleterious rather than strongly beneficial and are quickly lost (12). Some soil-acquired mutations were retained when soil-exposed populations were introduced to media (Fig. S2B), but the frequency of these mutations never rose above what was achieved by 60 dpi in soil, and more commonly fell below detection (Fig. S4). Moreover, in support of our prediction that media incubation would result in a shift in dominant mutations, TSB-acquired mutations equaled or outnumbered soil-acquired mutations at nearly every time point in all 12 soil-to-media populations (Fig. S2B). This suggests that a faster doubling time and the corresponding accumulation of ~140 media-based generations quickly overpowered the genetic signature acquired from time spent in soil.

**Fig 5:**
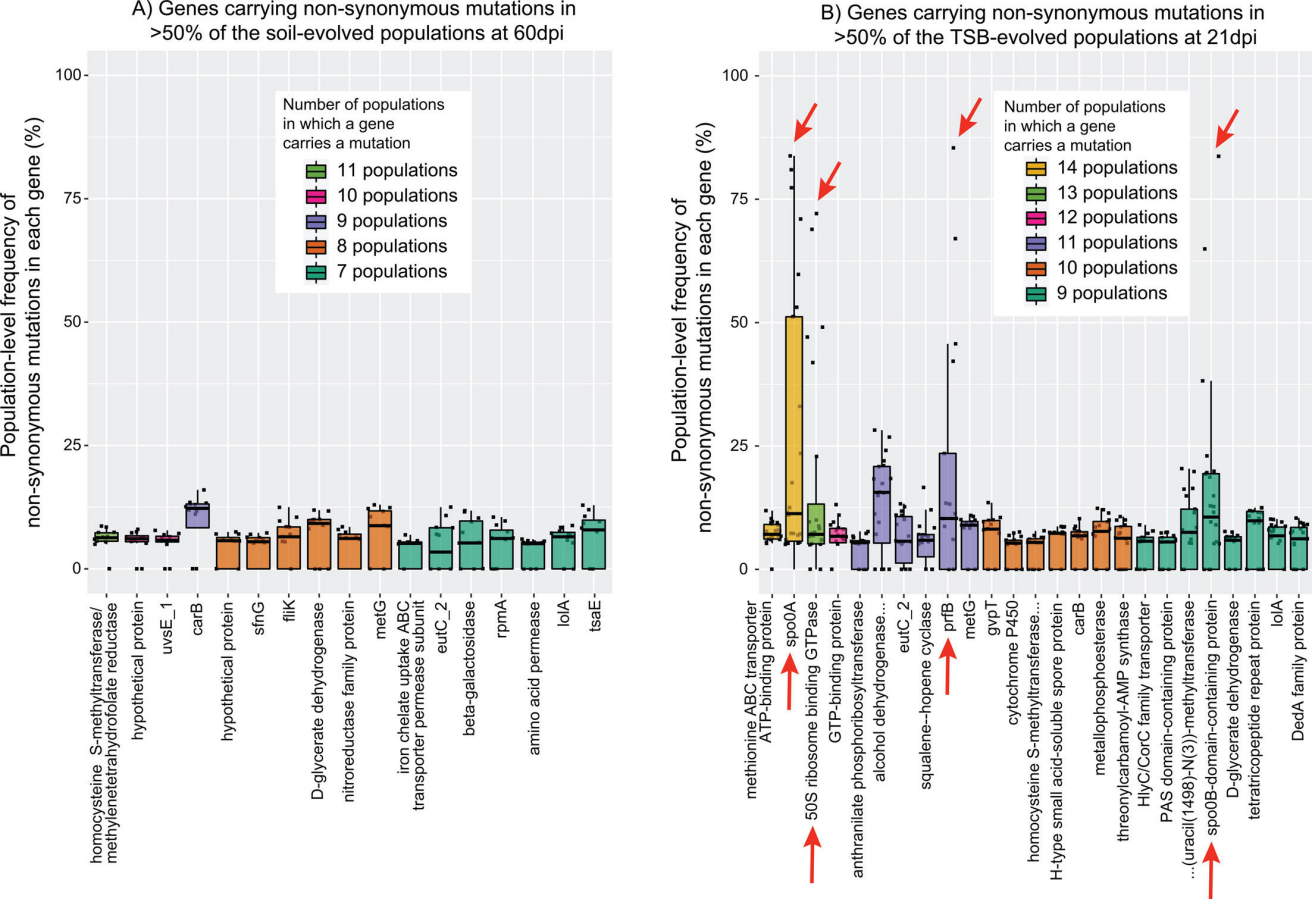
Mutation parallelism in soil- and TSB-evolved populations. (**A**) Population-level frequencies of non-synonymous mutations in genes displaying parallelism in the 60 dpi soil-evolved populations. (**B**) Population-level frequencies of non-synonymous mutations in genes displaying parallelism in the 21 dpi TSB-evolved populations. Each point represents a single mutation in a single population, and boxplots represent the distribution in the frequency of all mutations detected in any population for the indicated gene. Red arrows in panel B highlight genes displaying notable parallelism across TSB-incubated populations, and these genes are discussed further in the text. Further information on each of the genes listed can be found in Table S3.

**Fig 6:**
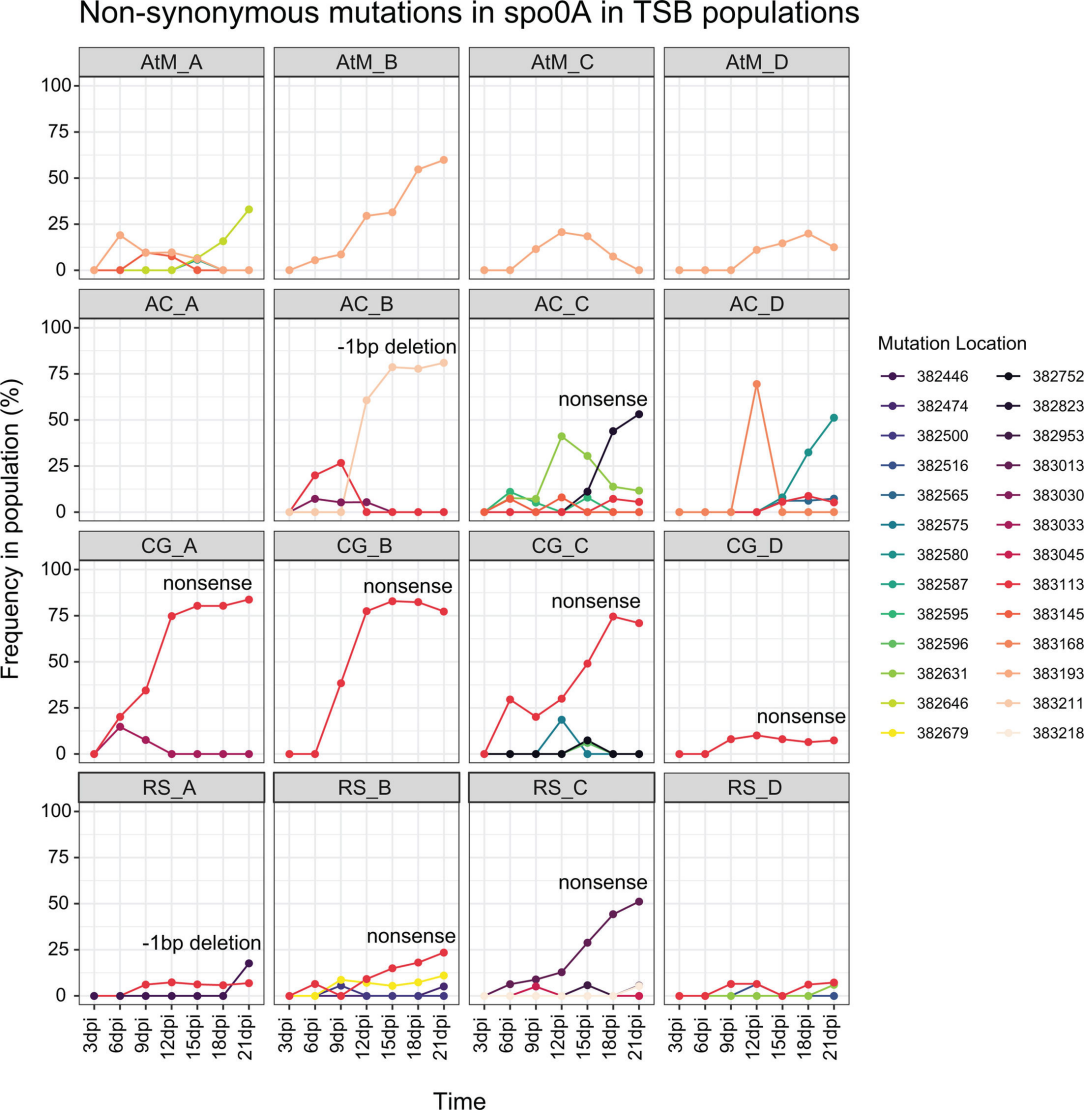
Population-level frequencies of non-synonymous mutations in the *spo0A* gene in each TSB-evolved population across time. Each color corresponds to a different mutation, as denoted by their bp location within contig 4 of the *de novo* assembled ancestral isolate genome. See Fig. S6 for more details. In-panel text highlights nonsense and frameshift mutations which achieved the highest frequency of all the *spo0A* mutations within their population at 21 days post-inoculation (dpi). Population type abbreviations: AtM = Ancestral-to-media, AC = Arboretum Cornfield, CG = Community Garden, RS = Russel E. Larson Agricultural Research Station.

Focusing on the final sampling points for soil and TSB, most mutated genes contained non-synonymous polymorphisms in only one or two populations (Fig. S3C and D). However, 18 genes in soil and 25 in TSB carried non-synonymous mutations in at least half of the populations (Table S5). The functional categories of these commonly mutated genes are listed in Table S5 and span a range of cellular traits and processes. It is possible that these genetic regions were more prone to modification, as is the case for short-motif repeat microsatellites or genes targeted by diversity-generating retroelements (63, 64). This may have been true for the six genes that were consistently mutated in both soil and TSB and arose again independently in the ancestral-to-media control populations (Table S5). However, if unique non-synonymous polymorphisms within a particular gene separately sweep to high frequencies in different replicate populations, such a pattern provides strong evidence that mutations in this gene have an adaptive benefit (12). We did not observe this pattern in soil-evolved populations (Fig. 5A), which made it difficult to address whether any of the observed mutations corresponded to soil abiotic selective pressures (prediction 2). It is also possible that some of the mutations observed in soil-exposed populations developed during the single day of growth they experienced in TSB while we were generating cells to inoculate into soil microcosms. In TSB-evolved populations, however, there were four genes displaying a strong pattern of parallelism, which encoded (i) the sporulation transcription factor Spo0A; (ii) a 50S ribosome-binding GTPase; (iii) peptide chain release factor 2 (PrfB); and (iv) a Spo0B domain-containing protein (Fig. 5B). It is particularly striking that for three of these four genes (*spo0A*, *prfB*, and the gene encoding the Spo0B domain-containing protein), mutations in these loci were never observed in any soil-evolved populations, appearing only after introduction to TSB and independently in the ancestral-to-media control populations. Especially for the sporulation-related genes, their lack of alteration in soil-based populations is understandable, given the importance of dormancy and rapidity of sporulation as a survival strategy in soil environments (65, 66). Within these genes, we also saw different mutations arising across time within single populations, with later mutations sometimes supplanting a previously dominant allele (Fig. 6 and Fig. S5), a pattern known as clonal interference (20). This indicates that multiple fitness peaks were present at different times throughout the experiment. Notably, for 9 of 16 populations, the highest frequency mutation in *spo0A* at 21 dpi was either a nonsense mutation or a frameshift mutation (Fig. 6), all but one of which occur in or before key functional domains of Spo0A and are likely to render this gene non-functional (Fig. S6, see additional detail in the Discussion). This implies that in nutrient-rich media, it was adaptive to disrupt the sporulation process, echoing findings from earlier studies (67, 68).

### Mutations detected within single isolates from endpoint populations

To validate mutations observed via population-level sequencing and assess mutation linkages, we randomly selected three isolates from each population at the final time point for whole genome sequencing. On average, isolates from TSB-evolved populations contained 4.79 mutations, with only one TSB-evolved isolate showing no genetic divergence from the ancestral isolate (Fig. 4B; Table S6). Different mutations were present in different isolates. In comparison, nearly half of the soil-evolved isolates (17 of 36) showed no genetic difference from the ancestral isolate, with an average of only 0.97 mutations (Fig. 4B Table S6). Moreover, many of the mutations in soil-evolved isolates were single bp insertions or deletions in mononucleotide repeat regions (Table S6), known to be hypermutation due to replication slippage (63). By contrast, mutations in TSB-evolved isolates were more varied in type (Table S6). This further reinforces our impression that the number of generations and corresponding evolutionary change had progressed less in soil compared to media.

In both sets of isolates, we detected mutations not detected by breseq from the population metagenomic data (Table S6), indicating that even deep metagenomic sequencing can miss rare genotypes. However, mutations at high population-level frequencies were only sometimes captured in one of the corresponding isolates, reinforcing the suggestion that both approaches should be used to reveal more detailed insights (15). For instance, for the TSB-evolved isolates, mutations co-located on the same genome often had highly dissimilar population-level frequencies (Table S6), indicating possible recombination with high-frequency alleles residing in several genotypic backgrounds (69). Less often, we also observed evidence of genetic hitchhiking, where some synonymous and intergenic mutations that were likely neutral achieved high frequencies (~40% to ~80%) in a particular population while co-located with a mutation in one of the four consistently mutated genes (Table S6, Fig S7). For example, in all three isolates from soil-to-media population AC-B, we observed three intergenic mutations co-located with a frameshift mutation in *spo0A* and a non-synonymous mutation in *prfB*, with each mutation present at ~80% frequency within the population (Table S6). Furthermore, the frequencies of all these mutations track nearly identically across time (Fig S7), suggesting that this single genotype swept to dominance in this population. It is possible that these high-frequency synonymous and intergenic mutations were themselves targets of positive selection, but more likely that selection on non-synonymous mutations drove several co-located mutations to higher frequencies together (12).

## DISCUSSION

In this study, we subjected a clonal population of *Priestia megaterium* to experimental evolution in sterile soil and liquid media. We aimed to determine whether in-soil survival is impacted by laboratory handling, if survival can be improved with pre-incubation in a target soil, and to identify the underlying genetic mutations shaping fitness changes. We detected rapid evolution when populations were passaged in nutrient-rich liquid media. In these media-exposed populations, we also observed strong fitness reductions in most soils and the potential disruption of sporulation abilities. Meanwhile, evolution in soil proceeded slowly, with limited generations of growth, resulting in subtle fitness trade-offs in unfamiliar soils rather than fitness gains in the target soil compared to the ancestral isolate.

Within <200 generations of incubation in TSB, there was a clear negative impact on microbial fitness in soil for both the ancestral-to-media and soil-to-media populations. Such trade-offs are not uncommon for microbes that experience two highly distinct environments. For instance, endosymbionts that evolve inside a host can display significant reductions in free-living fitness (70), and similar fitness trade-offs develop for *Bacillus* populations, which adapt to colonize plant rhizospheres (71, 72). The mechanisms driving these trade-offs are proposed to be either (i) mutation decay, defined as disuse and a lack of selection maintaining traits important for survival away from the host or (ii) antagonistic pleiotropy, driven by positive selection on traits that improve fitness near or within the host but have a direct negative impact on fitness away from the host (70). Similarly, microbes of interest for use as agricultural inoculants would experience both nutrient-rich, homogeneous media for production purposes and heterogeneous soil environments after inoculation, and it is clear that exposure to nutrient-rich media substantially impacts adaptation to soil environments.

When considering the genetic underpinnings of fitness changes in our media-evolved populations, it is notable that mutations in the same four genes swept to high frequencies within multiple replicate populations. There was a minimum of ~10^6^ individual cells after each daily culture dilution, so it is extremely unlikely that any mutation would rise to such frequencies in so few generations by random genetic drift alone (73), suggesting that these mutations were beneficial in the media environment and subject to positive selection (12). This implies that fitness trade-offs may have been driven more by antagonistic pleiotropy than mutational decay. Of these four gene products, two are involved in protein translation: the 50S ribosome GTPase is involved in ribosome biogenesis and assembly, and PrfB triggers the hydrolysis and release of translated peptide chains (74). While it is not straightforward to imagine what advantage might be conferred by mutations in these genes, it has been previously noted that beneficial mutations often occur in core metabolic genes due to wide-ranging pleiotropic effects (75).

More striking was the set of mutations occurring in the gene for the master transcriptional regulator of sporulation, *spo0A*. In its active, phosphorylated form, Spo0A~P binds to DNA sequences containing an “0A-box” (5′-TGTCGAA-3′), thereby directly or indirectly influencing the transcription of hundreds of genes to coordinate sporulation (76). Another commonly mutated gene, the Spo0B domain-containing protein, may also be involved in this process. Spo0B is a phosphotransferase that activates Spo0A (77), and the Spo0B-mediated phosphorylation of Spo0A could, in theory, be influenced by interaction with other factors or proteins (78). Our Spo0B domain-containing protein has a 14-residue region with homology to Spo0B, and there could be some interaction between the two. If so, this might influence the amount of active Spo0A~P, but this is mere speculation and beyond the scope of this study to determine. Sporulation is a lengthy, energetically costly, and irreversible process. Yet, this extreme measure would rarely be needed with frequent propagation in nutrient-rich TSB, so it would be beneficial to allocate this energy to cellular growth instead (79). Supporting this, several of the highest-frequency *spo0A* polymorphisms were nonsense or frameshift mutations occurring in or before the two key functional domains of Spo0A: (i) the response regulatory domain, which contains the site of phosphorylation and (ii) the helix-turn-helix domain, which is responsible for DNA binding (80). Such mutations are likely to generate non-functional versions of Spo0A, and these often out-competed other non-synonymous *spo0A* mutations within a single population. There is prior evidence of sporulation-deficient phenotypes arising during the experimental evolution of *Bacillus subtilis* in liquid media (68, 81), although the genetic basis of this phenotype is traced to other sporulation-related genes rather than *spo0A* (67). From a general standpoint, our findings agree with other evolution studies reporting mutations in transcriptional regulators or other transcriptional processes, as such changes can efficiently disable costly cellular processes (15, 82, 83). More specifically, these findings suggest that sporulation abilities may have high genetic instability in liquid media.

Such instability had strong implications for in-soil fitness in our study. *spo0A* mutants are known to be defective in their ability to sporulate and to form biofilms (84), and it has been repeatedly demonstrated that such deficiencies lead to worse survival outcomes in soil or on plant roots (85–88). It is, therefore, noteworthy that our observed in-soil fitness decreases were very often coupled with high-frequency *spo0A* mutations. Genome editing tools for *P. megaterium* have recently advanced (89), and an intriguing avenue for further study would be to engineer these individual *spo0A* mutations into the ancestral isolate and assess their impact on sporulation, biofilm formation, and in-soil fitness. We note that selection against sporulation in liquid media may be counterbalanced to some degree in agricultural inoculant development pipelines, as spores are often specifically generated for inclusion in inoculant formulations (90). Nonetheless, sporulation deficiencies could affect the efficiency of spore production for agricultural purposes. In addition, even populations that did not contain a high-frequency *spo0A* mutation still demonstrated decreased fitness in soil. This suggests that antagonistic pleiotropy was widespread among TSB-acquired mutations and not limited to *spo0A*. In either case, care should be taken to reduce the number of *in vitro* generations experienced by plant growth-promoting microbes during screening and formulation to preserve their fitness in soil environments. For instance, this could be achieved by maintaining glycerol stocks of the original soil isolates and returning to these stocks for subsequent inoculant production or using more nutrient-limited media for the bulk growth of inoculants.

By contrast, fitness changes in the soil-incubated populations were more subtle. In particular, there was no fitness improvement in the incubation soil compared to the ancestral isolate. We suspect that the number of generations experienced by our soil-exposed populations was quite limited, and it is also possible that a portion of the populations simply sporulated and therefore avoided selective pressures soon after inoculation into the soil microcosms. Both of these factors may have contributed to the limited fitness changes we observed. Our findings contrast with several studies in which soil-evolved isolates outperformed the ancestral isolate in non-sterile soil fitness tests despite a shorter soil incubation (48 d instead of 60 d) (23, 29). However, an estimate of the number of generations experienced by the *Pseudomonas fluorescens* SBW25 populations in these studies was not provided, so more evolutionary time may have passed. We did observe a marginal decrease in the fitness of our soil-evolved populations compared to the ancestral isolate in unfamiliar soils, which can be explained if mutations with seemingly neutral or positive effects on fitness in one soil had deleterious effects in a different soil. A similar trade-off between different soils was noted by Yates et al. (25), where the fitness of a *Pseudomonas* isolate improved after 92 days of incubation in a novel soil, to the detriment of its fitness in the soil from which it was initially isolated. Our observed trade-off held even after ~150 generations in liquid media, implying that an evolutionary footprint from the time a population spent in soil was somewhat retained despite laboratory handling. This suggests that adaptations to native soil may remain in laboratory-maintained microbial isolates, impacting their success in other soils.

Genetic changes in the soil-incubated populations were also limited. No mutations swept to frequencies higher than ~25%, and there were no strong signatures of mutational parallelism as observed in media-incubated populations. However, our soil-incubated populations likely underwent far fewer generations than the TSB-incubated populations despite being evolved for a longer absolute time, even when accounting for probable population turnover after our *P. megaterium* populations plateaued. A similar population plateau was observed in another soil experimental evolution study with a non-spore-forming taxon (26), which indicates that a key challenge for such experiments is ensuring that populations are actively multiplying whether or not the focal microbe can go dormant. Future experiments can include plants in soil microcosms, as carbon resources via plant root exudates could improve bacterial growth and speed evolutionary processes. Without comparable generations experienced in the soil- vs. media-based incubations, generation time was a likely source of differences in the degree of evolutionary change. Strategies to achieve more in-soil generations could include inoculating a lower initial concentration of cells, inoculating a larger initial volume of sterile soil, or performing more frequent population bottlenecks into freshly sterilized soil. This final approach would more closely replicate the daily bottlenecks of classic experimental evolution studies but would compromise how well it replicates real-world conditions.

In future experiments where generational time for soil- and media-based populations is more comparable, it is worth exploring whether there are inherent differences in evolutionary patterns in soil. Because of spatial heterogeneity, populations expanding through the soil matrix will likely encounter different conditions from one mm of soil to the next (19, 91), complicating and potentially diluting the influence of particular selective pressures. Furthermore, at the edge of a population expanding through space, mutations occurring in “frontier” cells can sometimes reach high frequencies in subsequently colonized territories in a stochastic process known as gene surfing (92). Such mutations may differ from one region of soil to another. Spatially variable soil conditions can also lead to spatially variable bacterial growth rates, meaning that bacteria of vastly different ages can co-exist (93) and that a cross-section of ancestors and descendants may be captured during population sequencing, further diluting signals of mutation sweeps. More generally, the spatial complexity of soil could result in multiple sub-populations separated across aggregates but loosely linked by dispersal, with each sub-population experiencing distinct selective pressures (94) and a higher vulnerability to stochastic processes due to their smaller size (92). Each of these processes could contribute to an overall lack of dominant alleles. While we thoroughly mixed our soil microcosms during construction and before sampling, it would be intriguing to leave the experimental soil undisturbed and sample several sub-locations in each microcosm to determine whether sweeps are occurring at a more localized level. We also note that because we removed resident microbes, our soil microcosms remain simplified compared to natural soil ecosystems. Interactions with other microbes can exert strong selective pressures on evolving populations (10, 95, 96) and speed genetic and phenotypic modification through mechanisms such as horizontal gene transfer (97), and further exploration of these biotic influences during soil-based evolution is warranted.

Taken together, our results indicate that evolutionary histories, particularly in laboratory media, impact real-world microbial fitness. We suggest limiting continuous propagation in media, maintaining stocks of the original isolate, and screening isolate fitness in various soils to improve inoculant outcomes and preserve microbial traits related to in-soil survival. While our observed discrepancies between soil- and media-based evolution could not be untangled from differences in generation time, we suggest that there are limits to extending microbial evolution patterns beyond their *in vitro* setting. There is a need for further soil-based studies to continue untangling the impact of this complex environment on microbial evolutionary dynamics.

## Data Availability

Raw sequence files from this study are available in the NCBI SRA database under BioProject accession numbers PRJNA928463 and PRJNA930757.

## SUPPLEMENTAL MATERIAL

Supplemental material aem.02085-24-s0001.pdf Figures S1 to S7 and Tables S1, S2, S4, and S5.

Table S3 aem.02085-24-s0002.xlsx Statistical tests associated with Fig. 2.

Table S6 aem.02085-24-s0003.xlsx Mutations detected in single isolates.

ASM does not own the copyrights to Supplemental Material that may be linked to, or accessed through, an article. The authors have granted ASM a non-exclusive, world-wide license to publish the Supplemental Material files. Please contact the corresponding author directly for reuse.
