# Supplementary material for: Evolving a plant-beneficial bacterium in soil vs. nutrient-rich liquid culture has contrasting effects on in-soil fitness
Source: Appl Environ Microbiol. 2025 Mar 11;91(4):e02085-24. doi: 10.1128/aem.02085-24 (PMC12016532; doi:10.1128/aem.02085-24)
Supplement: Supplemental material — Figures S1 to S7 and Tables S1, S2, S4, and S5. [file aem.02085-24-s0001.pdf]

SUPPLEMENTAL FIGURES AND TABLES

Figure S1: Population size of *P. megaterium* in the soil microcosms across time during the soil incubation according to CFU counts on TSA. Shown is the mean±se for the four replicate microcosms of each soil type (AC= Arboretum Cornfield, CG = Community Garden, RS = Russel E. Larson Agricultural Research Station). Population sizes in soil at day 0 (the initial inoculation) and day 30 (the re-inoculation after the population bottleneck) are estimates based on the number of CFUs added to each microcosm.

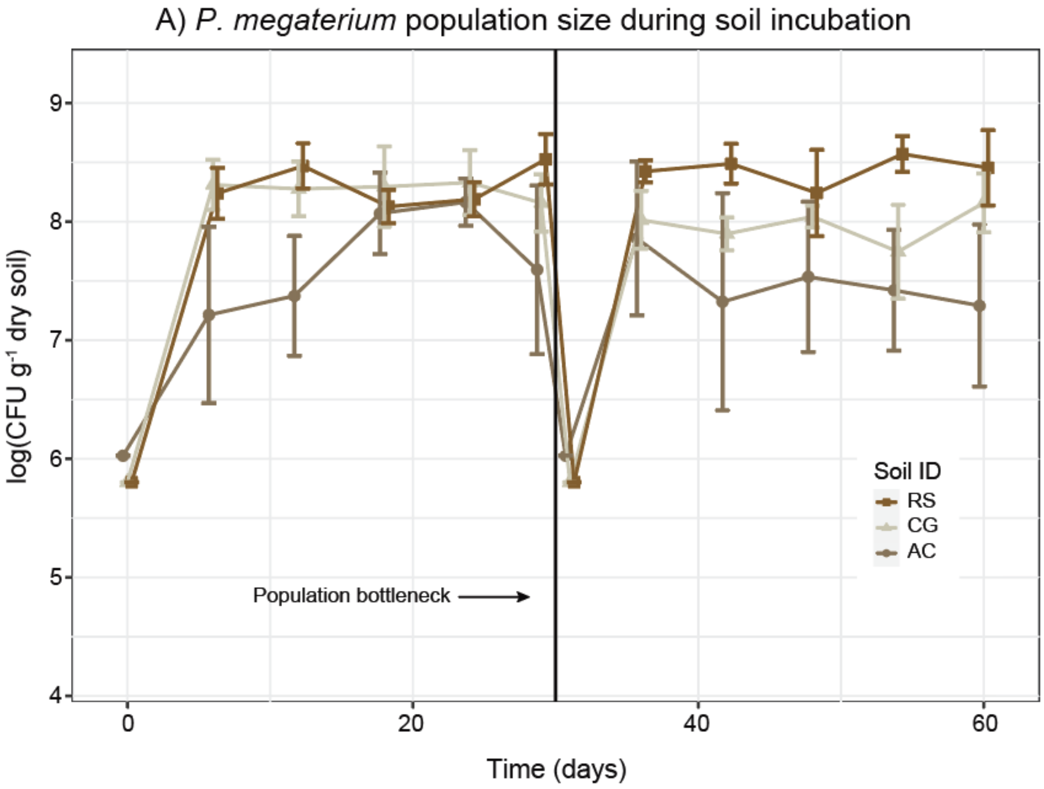

Figure S2: Retention of non-synonymous mutations across time for **(A)** soil-exposed populations and **(B)** TSB-exposed populations. Shown is the percentage of mutations that have or have not been detected before within a particular population at each time point, averaged across the four replicate microcosms of the indicated treatment. The average number of non-synonymous mutations detected at each time point are also displayed. Soil type abbreviations: AC= Arboretum Cornfield, CG = Community Garden, RS = Russel E. Larson Agricultural Research Station, AtM = Ancestral-to-media.

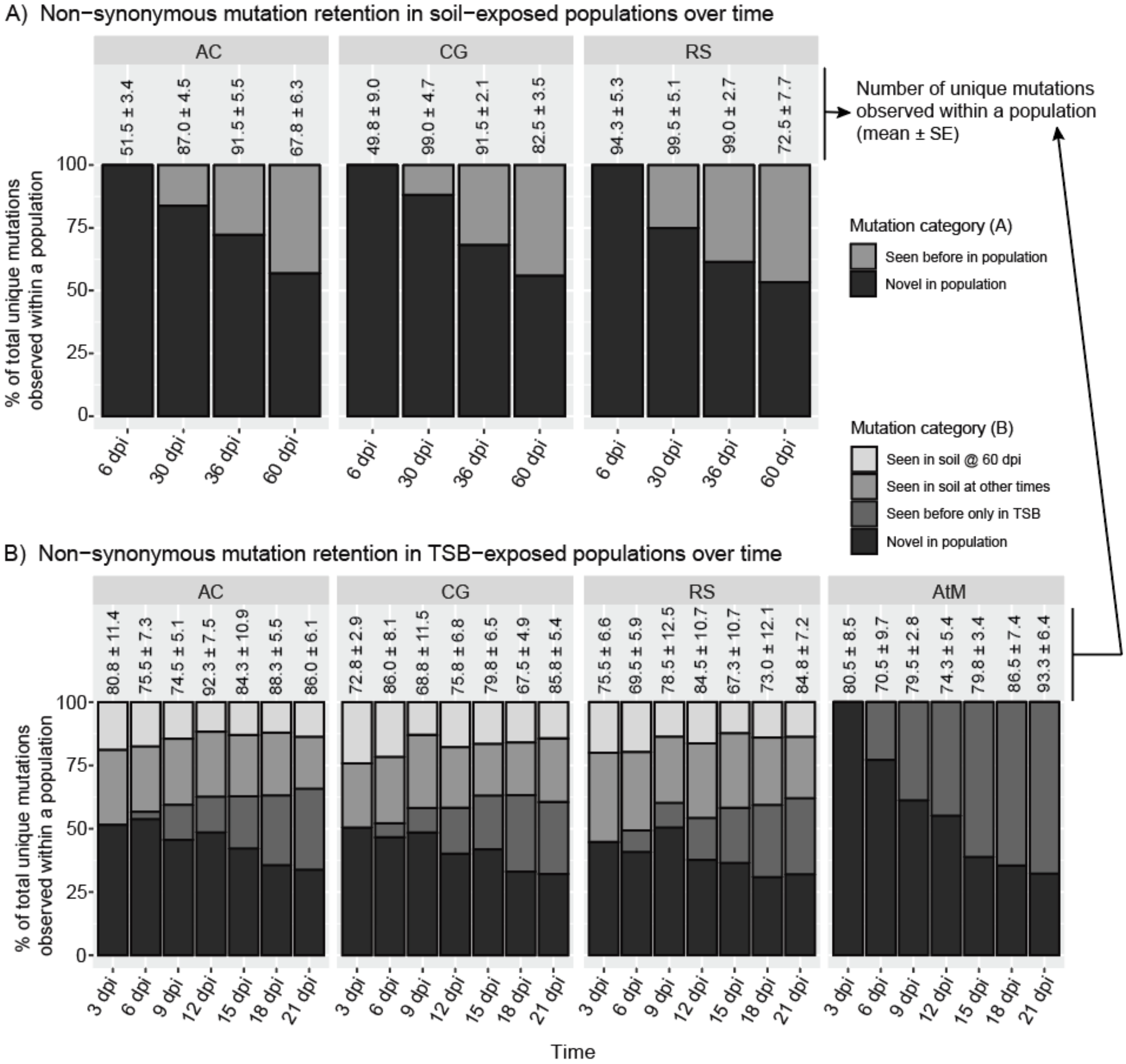

<u>Figure S3</u>: Most non-synonymous mutations are detected at only one time point, and most mutated genes have developed a non-synonymous mutation only in 1-2 populations. **(A)** Histogram tallying the number of time points at which a non-synonymous mutation appears in the populations evolving in soil and **(B)** the populations evolving in TSB. Each bar represents the count for a single population. **(C)** Histogram of the number of 60 dpi soil-evolved populations in which a gene carries a non-synonymous mutation. **(D)** Histogram of the number of 21 dpi TSB-evolved populations in which a gene carries a non-synonymous mutation.

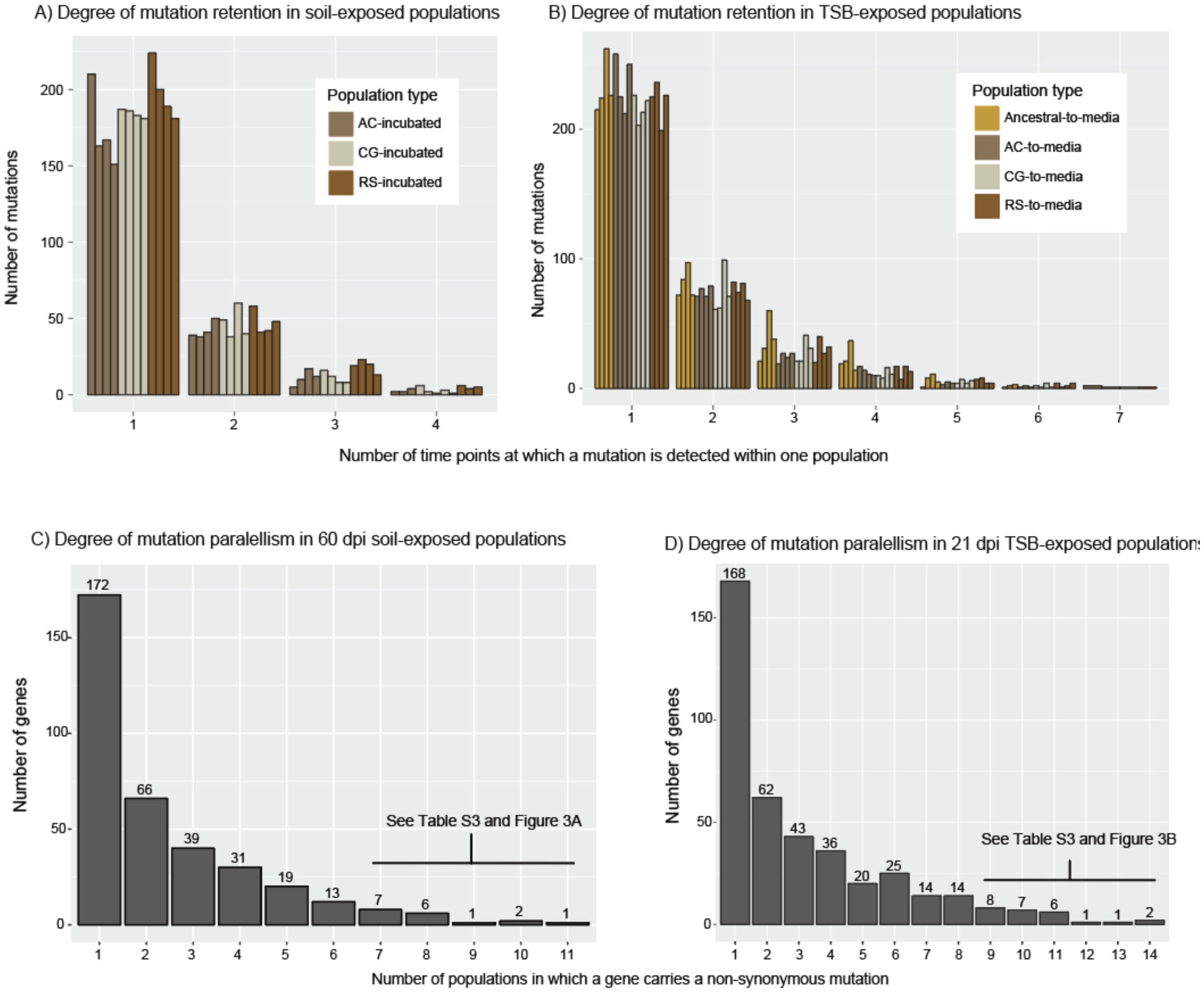

<u>Figure S4:</u> Frequency across time of non-synonymous mutations detected at 60 days post inoculation (dpi) in soil-evolved populations, once that population was introduced to TSB. Only mutations with >10% frequency at 60 dpi are shown for simplicity. Each color represents a distinct single mutation, and the legend notes the location of that mutation within the indicated contig in the de novo assembled ancestral *P. megaterium* genome (e.g. NODE01_0070240 denotes a mutation occurring at base pair 70240 within contig 1 of the genome). Note the y-axis scale runs from 0% to 50% rather than 100%. Each panel displays data from a single replicate population. Soil type abbreviations: AC= Arboretum Cornfield, CG = Community Garden, RS = Russel E. Larson Agricultural Research Station.

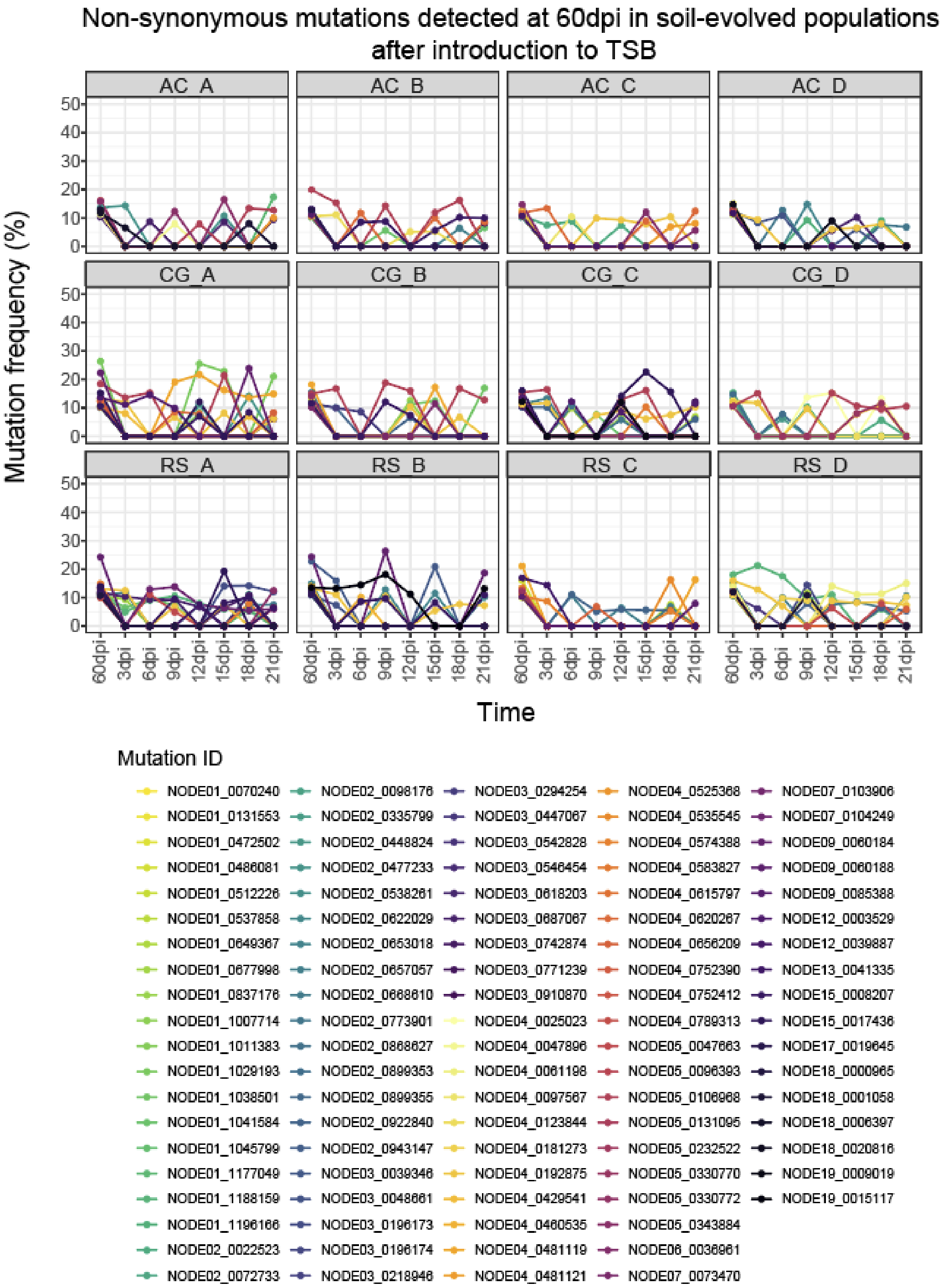

Figure S5: Population-level frequencies of non-synonymous mutations in A) the spo0B domain-containing protein gene; B) the prfB gene; and C) a 50S ribosome-binding GTPase gene in each individual TSB-evolved population out to 21 days post inoculation (dpi). Mutation ID keys are distinct for each panel. Different colors represent different individual mutations, and mutation locations within the indicated contig of the *de novo* assembled ancestral *P. megaterium* genome are listed (e.g. NODE04_0573618 denotes a mutation occurring at base pair 573618 within contig 4 of the genome). Population type abbreviations: AtM = Ancestral-to-media, AC= Arboretum Cornfield, CG = Community Garden, RS = Russel E. Larson Agricultural Research Station.

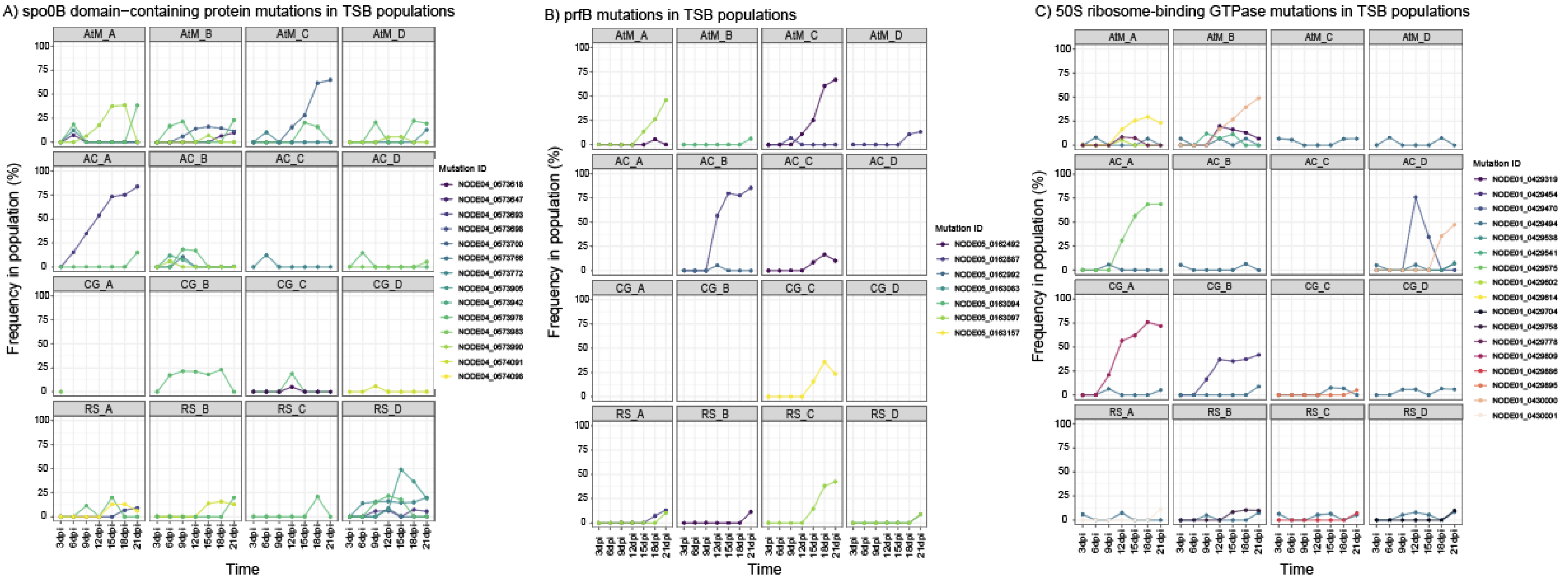

<u>Figure S6:</u> Location of mutations displayed in Figure 5 within the spo0A gene. Marks below each mutation indicate the number of different populations in which that particular mutation was observed during at least one time point. Red marks further indicate that this mutation was the highest frequency mutation in the spo0A gene in that population at 21dpi. Stars further indicate that this mutation had achieved at least 50% frequency in that population. The lowermost text in each line denotes the forward read (3' → 5') while the middle line of text denotes the complementary reverse read and coding sequence (5' → 3'). The corresponding amino acid translation is listed above. Base pair positions within contig 4 of the *de novo* assembled ancestral *P. megaterium* genome are marked at either line end. Blue highlighted amino acids correspond to the response regulator domain of the Spo0A protein. Yellow highlighted amino acids correspond to the helix-turn-helix DNA binding domain of the Spo0A protein. Mutations highlighted blue indicate single base pair deletions, while mutations highlighted red indicate nonsense mutations. All other non-synonymous mutations are highlighted grey.

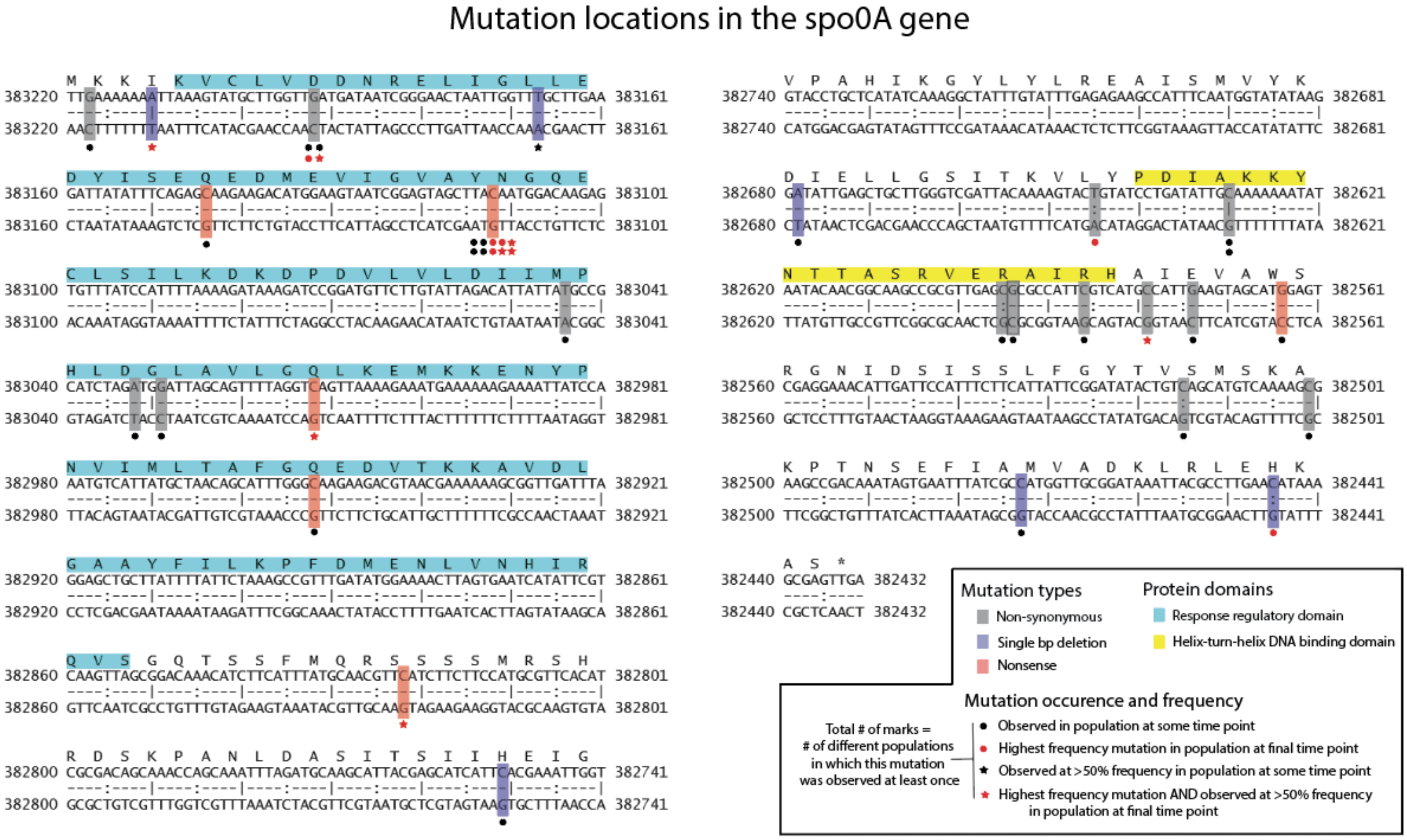

<u>Figure S7</u>: Population-level frequency across time of mutations present in single isolates sequenced after 21 days in TSB, demonstrating potential genetic hitchhiking events. These isolates were selected for display because they contained synonymous or intergenic mutations with high population-level frequencies similar to co-occurring non-synonymous mutations in *spo0A*. Each line represents a single unique mutation. Lines are colored by mutation type. For non-synonymous mutations, the gene in which the mutation occurs is labelled with an arrow. Further information on each mutation can be found in Table S4 under the corresponding isolate entry.

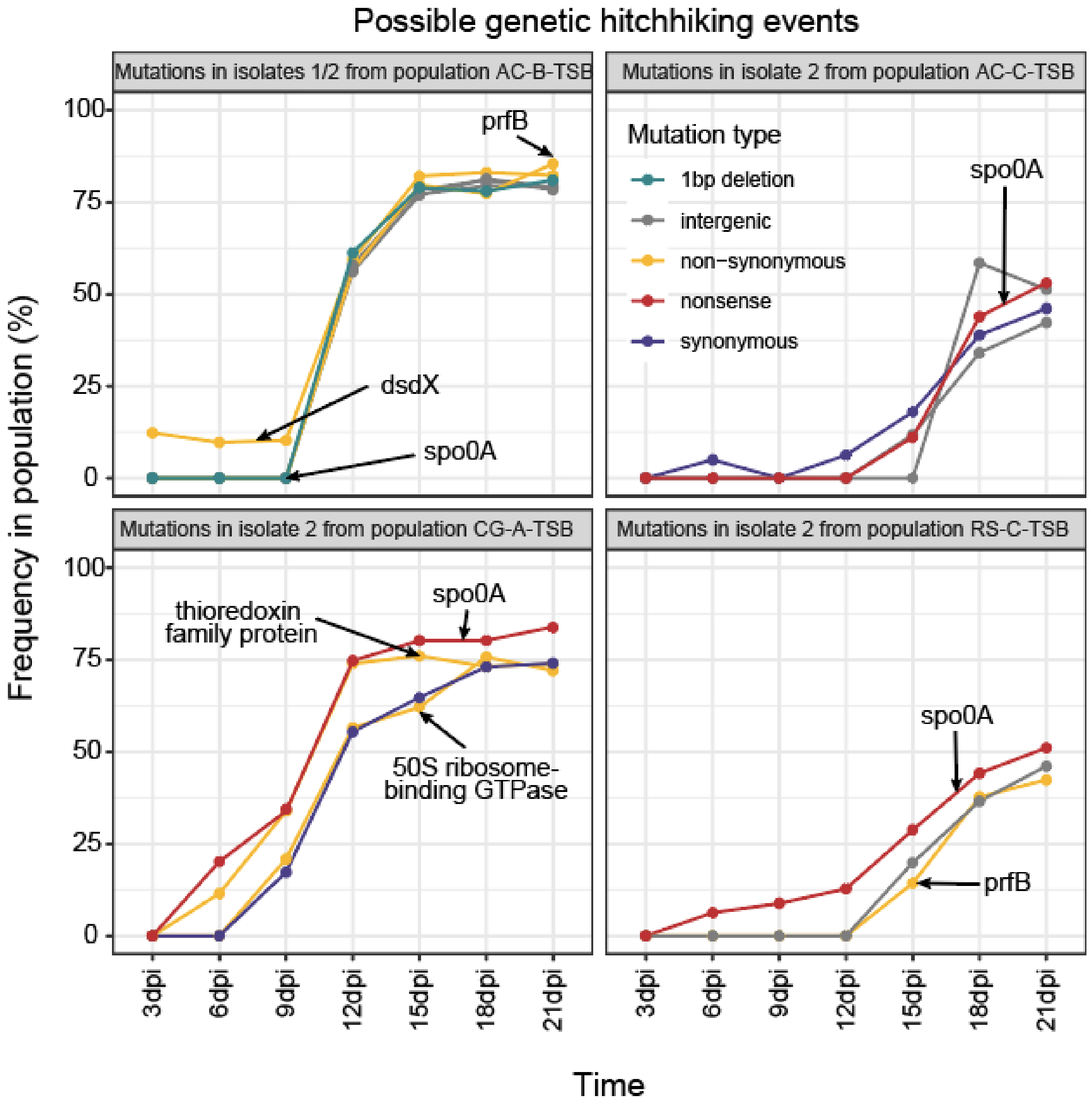

<u>Table S1:</u> Soil properties of the thrice-autoclaved AC, CG, and RS soils in which *P. megaterium* was incubated for 60 days (sterilized), and the unaltered AC, CG, RS, and CCC soils used for the soil fitness tests (unsterilized). pH was determined in a 1:1 water to soil suspension. Organic matter was determined by mass loss on combustion. Water content was determined by mass loss after 24h of drying at 70ºC. Nitrate content was determined by specific ion electrodes. All other nutrient contents were determined with Mehlich 3 extractables.

<table>
<tr><th rowspan="2">Soil Property</th><th colspan="7">Soil Name (Soil ID)</th></tr>
<tr><th colspan="2">Arboretum Cornfield (AC)</th><th colspan="2">Community Garden (CG)</th><th colspan="2">Russel E. Larson Agricultural Research Station (RS)</th><th>Cover Crop Cocktail site (CCC)</th></tr>
<tr><td>Coordinates</td><td colspan="2">40.809430, -77.879467</td><td colspan="2">40.811295, -77.847633</td><td colspan="2">40.711733, -77.935808</td><td>40.722618, -77.926866</td></tr>
<tr><td>Land Use</td><td colspan="2">Monoculture corn</td><td colspan="2">Fallow field</td><td colspan="2">Monoculture soybean</td><td>Rye cover crop</td></tr>
<tr><td rowspan="2">Soil type</td><td colspan="2">Hagerstown silt loam</td><td colspan="2">Hagerstown silty clay loam</td><td colspan="2">Murrill channery silt loam</td><td>Murrill channery silt loam</td></tr>
<tr><th>Sterilized</th><th>Unsterilized</th><th>Sterilized</th><th>Unsterilized</th><th>Sterilized</th><th>Unsterilized</th><th>Unsterilized</th></tr>
<tr><td>pH</td><td>6.68</td><td>7.05</td><td>5.82</td><td>5.57</td><td>5.83</td><td>4.9</td><td>6.49</td></tr>
<tr><td>Organic matter (%)</td><td>9.04</td><td>6.89</td><td>2.95</td><td>3.01</td><td>3.83</td><td>4.2</td><td>2.2</td></tr>
<tr><td>Water content (%)</td><td>46.8%</td><td>41.8%</td><td>21.2%</td><td>31.0%</td><td>24.2%</td><td>34.8%</td><td>32.4%</td></tr>
<tr><td>Nitrate (ppm)</td><td>148.5</td><td>126.1</td><td>42</td><td>53.9</td><td>72.25</td><td>95.9</td><td>17.5</td></tr>
<tr><td>Ammonium (ppm)</td><td>242</td><td>2.2</td><td>67.9</td><td>1.4</td><td>81.5</td><td>0.8</td><td>1.3</td></tr>
<tr><td>Phosphorous (ppm)</td><td>272</td><td>149</td><td>74</td><td>34</td><td>189</td><td>75</td><td>45</td></tr>
<tr><td>Potassium (ppm)</td><td>274</td><td>228</td><td>114</td><td>76</td><td>197</td><td>203</td><td>207</td></tr>
<tr><td>Magnesium (pm)</td><td>205</td><td>147</td><td>213</td><td>160</td><td>66</td><td>54</td><td>114</td></tr>
<tr><td>Calcium (ppm)</td><td>3053.9</td><td>3189.8</td><td>1308.3</td><td>1140.2</td><td>620.8</td><td>529.3</td><td>1049.4</td></tr>
<tr><td>Zinc (ppm)</td><td>14.3</td><td>12.1</td><td>2.9</td><td>3.8</td><td>1.6</td><td>2.7</td><td>4.1</td></tr>
<tr><td>Copper (ppm)</td><td>2.4</td><td>5.7</td><td>2</td><td>4.5</td><td>1.6</td><td>3</td><td>5.5</td></tr>
<tr><td>Sulfur (ppm)</td><td>62.7</td><td>16.6</td><td>38.3</td><td>9.2</td><td>59.2</td><td>12.4</td><td>10.4</td></tr>
</table>

<u>Table S2:</u> Quality of the *de novo* assembled ancestral *P. megaterium* genome used as a reference for subsequent mutation analysis.

**Quality metric**	**Ancestral *P. megaterium* genome**
**# contigs (>= 0 bp)**	68
**# contigs (>= 1000 bp)**	29
**# contigs (>= 5000 bp)**	23
**# contigs (>= 10000 bp)**	22
**# contigs (>= 25000 bp)**	18
**# contigs (>= 50000 bp)**	14
**Total length (>= 0 bp)**	5488789
**Total length (>= 1000 bp)**	5476473
**Total length (>= 5000 bp)**	5463024
**Total length (>= 10000 bp)**	5455218
**Total length (>= 25000 bp)**	5396810
**Total length (>= 50000 bp)**	5236558
**# contigs**	68
**Largest contig**	1205600
**Total length**	5488789
**GC (%)**	37.74
**N50**	937557
**N90**	93007
**L50**	3
**L90**	9
**# N's per 100 kbp**	0
**Average coverage**	110.242

Table S3: Results of ANOVA and Tukey's HSD, testing differences in population density between different fitness test microcosms at each time point (0 to 15 days post inoculation (dpi)), as displayed in **Figure 2**. Only populations inoculated into the same soil type are compared. Soil type abbreviations: AC= Arboretum Cornfield, CG = Community Garden, RS = Russel E. Larson Agricultural Research Station, CCC = Cover Crop Cocktail site.

See file: "TableS3_FitnessTest_Stats.xlsx"

<u>Table S4:</u> Statistical test values for **Figure 3**. Independent Welch two-sample t-tests were used to test the hypothesis that evolved populations had significantly different population sizes compared to the ancestral isolate (for soil-incubated populations) or ancestral-to-media populations (for soil-to-media populations) at the final time point. P-value symbols: < 0.1 = ~ ; <0.05 = *; <0.005 = **

**Comparison**	**Soil**	**t**	**df**	**p-value**
Ancestral vs. AC-incubated	AC (home)	0.23501	5.8121	0.8223
	CCC (away)	-3.3333	5.5864	**0.01752 ***
Ancestral vs. CG-incubated	CG (home)	-1.9123	3.4196	0.1404
	CCC (away)	2.3222	4.2394	0.07724 ~
Ancestral vs. RS-incubated	RS (home)	-2.6777	5.1712	**0.04247 ***
	CCC (away)	2.2102	4.7054	0.08236 ~
Ancestral-to-media vs. AC-to-media	AC (home)	-0.19715	5.6573	0.8506
	CCC (away)	-5.0522	5.0955	**0.003722 ****
Ancestral-to-media vs. CG-to-media	CG (home)	0.16427	4.7416	0.8763
	CCC (away)	6.45	3.7209	**0.003801 ****
Ancestral-to-media vs. RS-to-media	RS (home)	-2.2504	5.4962	0.06944 ~
	CCC (away)	5.6419	5.9658	**0.001355 ****

<u>Table S5:</u> List of the genes that housed non-synonymous in over half of the soil-evolved populations after 60 days and over half of the TSB-evolved populations after 21 days, as displayed in Figure 3. The breseq-assigned annotation and functional information for each gene is included. Bolded genes were present in this list for both soil-evolved and TSB-evolved populations. For these genes only, the number of ancestral-to-media replicate populations that housed mutations in these genes are highlighted with "+" symbols.

**SOIL-EVOLVED POPULATIONS AFTER 60 DAYS**				
**Gene ID**	**Gene annotation**	**Number of populations**	**General functional category**	**Citation**
**gene_01932**	**bifunctional homocysteine S-methyltransferase/ methylenetetrahydrofolate reductase**	11	Amino acid biosynthesis and/or metabolism	Deng, Mu [85]
gene_00615	hypothetical protein	10	Unknown	-
uvsE_1	UV DNA damage repair endonuclease UvsE	10	UV and desiccation tolerance	Goosen and Moolenaar [86], [87]
**carB**	**carbamoyl-phosphate synthase large subunit**	9	Amino acid biosynthesis and/or metabolism	Wang, Jiang [88]
gene_00271	hypothetical protein	8	Unknown	-
sfnG	dimethyl sulfone monooxygenase SfnG	8	Nutrient utilization/ metabolism (S)	Wicht [89]
gene_03543	flagellar hook-length control protein FliK	8	Motility	Shibata, Takahashi [90]
**gene_04312**	**D-glycerate dehydrogenase**	8	Amino acid biosynthesis and/or metabolism	Ali, Shigeta [91], [92]
gene_04366	nitroreductase family protein	8	Nutrient utilization/ metabolism (N)	Carles, Donnadieu [93]
**metG**	**methionine--tRNA ligase**	8	Translation processes	Deniziak and Barciszewski [94]
gene_02126	iron chelate uptake ABC transporter family permease subunit	7	Nutrient uptake (Fe)	Köster [95]

**eutC_2**	**ethanolamine ammonia-lyase subunit EutC**	7	Nutrient utilization/ metabolism (C/N)	Costa and Escalante-Semerena [96]
gene_03183	beta-galactosidase	7	Nutrient utilization/ metabolism (C)	He, Priebe [97]
rpmA	50S ribosomal protein L27	7	Translation processes*	Pidutti, Federici [98]
gene_05221	amino acid permease	7	Amino acid uptake	Hosie and Poole [99]
**gene_05406**	**outer membrane lipoprotein carrier protein LolA**	7	Installation of cell outer membrane components	Kaplan, Greene [100]
tsaE	tRNA (adenosine(37)-N6)-threonylcarbamoyltransferase complex ATPase subunit type 1 TsaE	7	Translation processes	Luthra, Swinehart [101], [102]
**TSB-EVOLVED POPULATIONS AFTER 21 DAYS**				
**Gene ID**	**Gene annotation**	**Number of populations**	**General functional category**	**Citation**
gene_02254	methionine ABC transporter ATP-binding protein	14	Amino acid uptake	Kadaba, Kaiser [103]
spo0A	sporulation transcription factor Spo0A	14	Sporulation initiation	Jiang, Shao [66]
gene_00478	50S ribosome-binding GTPase	13	Ribosome biogenesis	Kaczanowska and Rydén-Aulin [63]
gene_03106	GTP-binding protein	12	Unknown	Boquet [104]
gene_01022	anthranilate phosphoribosyltransferase	11	Amino acid biosynthesis and/or metabolism	Lambrecht and Downs [105]
gene_01154	alcohol dehydrogenase catalytic domain-containing protein	11	Cell metabolism	Agarwal, Gitaitis [106]
**eutC_2**	**ethanolamine ammonia-lyase subunit EutC**	11 (AtM = +++)	Nutrient utilization/ metabolism (C/N)	Costa and Escalante-Semerena [96]
gene_02929	squalene--hopene cyclase	11	Biosynthesis of cell membrane components	Wendt, Lenhart [107]

prfB	peptide chain release factor 2	11	Translation processes	Kaczanowska and Rydén-Aulin [63]
**metG**	**methionine--tRNA ligase**	11 (AtM = +++)	Translation processes	Deniziak and Barciszewski [94]
gene_00709	gas vesicle protein GvpT	10	Buoyancy in water	Eppinger, Bunk [61]
gene_00754	cytochrome P450	10	Cell energy generation (electron transport chain)	Munro, Leys [108]
**gene_01932**	**bifunctional homocysteine S-methyltransferase/methylenetetrahydrofolate reductase**	10 (AtM = ++++)	Amino acid biosynthesis and/or metabolism	Deng, Mu [85]
gene_03013	H-type small acid-soluble spore protein	10	Spore protein	Setlow, Sun [109]
**carB**	**carbamoyl-phosphate synthase large subunit**	10 (AtM = +++)	Amino acid biosynthesis and/or metabolism	Wang, Jiang [88]
gene_04058	metallophosphoesterase	10	Cell metabolism	[110]
gene_04506	threonylcarbamoyl-AMP synthase	10	Translation processes	Luthra, Paranagama [111]
gene_00996	HlyC/CorC family transporter	9	Magnesium transport	Jin, Huang [112]
gene_02785	PAS domain-containing protein	9	Unknown	Hefti, Françoijs [113]
gene_03919	16S rRNA (uracil(1498)-N(3))-methyltransferase	9	Ribosome biogenesis	Zhang, Wan [114]
gene_04015	Spo0B domain-containing protein	9	Unknown, possibly involved in sporulation	Varughese, Zhou [67]
**gene_04312**	**D-glycerate dehydrogenase**	9 (AtM = ++++)	Amino acid biosynthesis and/or metabolism	Ali, Shigeta [91], [92]
gene_04402	tetratricopeptide repeat protein	9	Unknown	Blatch and Lässle [115]

**gene_05406**	**outer membrane lipoprotein carrier protein LolA**	9 (AtM = ++)	Installation of cell outer membrane components	Kaplan, Greene [100]
gene_05563	DedA family protein	9	Membrane protein	Doerrler, Sikdar [116]

Table S6: Mutations detected in each single isolate sequenced at 60 days post inoculation (dpi) for the soil-evolved populations or 21 dpi for the TSB-evolved populations. Three individual isolates were sequenced from each experimental population. Isolates with no detected mutations are colored gray. If a mutation was intergenic, the two most closely located genes are listed for the Gene ID and Gene Annotation. For population-level frequencies, if a particular mutation was not detected in the metagenomic sequencing data for that population, "ND" (not detected) is listed. Soil type abbreviations: AC= Arboretum Cornfield, CG = Community Garden, RS = Russel E. Larson Agricultural Research Station, AtM = Ancestral-to-media.

See file: "TableS6_Single_isolate_mutations.xslx"

REFERENCES

1. Mitter, E.K., et al., *Rethinking Crop Nutrition in Times of Modern Microbiology: Innovative Biofertilizer Technologies.* Front. Sustain. Food Syst., 2021. **5**: p. 606815.
2. Raymaekers, K., et al., *Screening for novel biocontrol agents applicable in plant disease management – a review.* Biol. Cont., 2020. **144**: p. 104240.
3. Bitterlich, M., et al., *Arbuscular mycorrhizas: a promising component of plant production systems provided favorable conditions for their growth.* Frontiers in Plant Science, 2018. **9**.
4. Imperiali, N., et al., *Relationships between root pathogen resistance, abundance and expression of Pseudomonas antimicrobial genes, and soil properties in representative Swiss agricultural soils.* Frontiers in plant science, 2017. **8**: p. 427.
5. Owen, D., et al., *Use of commercial bio-inoculants to increase agricultural production through improved phosphrous acquisition.* Appl. Soil Ecol., 2015. **86**: p. 41-54.
6. Salomon, M., et al., *Global evaluation of commercial arbuscular mycorrhizal inoculants under greenhouse and field conditions.* Appl. Soil Ecol., 2022. **169**: p. 104225.
7. Thilakarathna, M.S. and M.N. Raizada, *A meta-analysis of the effectiveness of diverse rhizobia inoculants on soybean traits under field conditions.* Soil Biol. Biochem., 2017. **105**: p. 177-196.
8. Kaminsky, L.M. and T.H. Bell, *Novel primers for quantification of Priestia megaterium populations in soil using qPCR.* Applied Soil Ecology, 2022. **180**: p. 104628.
9. Duffy, B.K. and G. Défago, *Controlling instability in gacS-gacA regulatory genes during inoculant production of Pseudomonas fluorescens biocontrol strains.* Applied and environmental microbiology, 2000. **66**(8): p. 3142-3150.
10. Liu, B., et al., *Natural Escherichia coli isolates rapidly acquire genetic changes upon laboratory domestication.* Microbiology, 2017. **163**(1): p. 22-30.
11. Voisard, C., et al., *Biocontrol of root diseases by Pseudomonas fluorescens CHA0: current concepts and experimental approaches.* Molecular ecology of rhizosphere microorganisms: biotechnology and the release of GMOs, 1994: p. 67-89.
12. Gómez, P., et al., *Local adaptation of a bacterium is as important as its presence in structuring a natural microbial community.* Nature Communications, 2016. **7**(1): p. 12453.
13. van Houte, S., et al., *Compost spatial heterogeneity promotes evolutionary diversification of a bacterium.* Journal of evolutionary biology, 2021. **34**(2): p. 246-255.
14. Yates, C.F., et al., *Rapid niche shifts in bacteria following conditioning in novel soil environments.* Functional Evology, 2022(00): p. 1-11.
15. Hughes, B.S., A.J. Cullum, and A.F. Bennett, *Evolutionary adaptation to environmental pH in experimental lineages of Escherichia coli.* Evolution: International Journal of Organic Evolution, 2007. **61**(7): p. 1725-1734.
16. Lawrence, D., et al., *Species interactions alter evolutionary responses to a novel environment.* PLoS biology, 2012. **10**(5): p. e1001330.
17. Vasse, M., S. Bonhoeffer, and A. Frenoy, *Ecological effects of stress drive bacterial evolvability under sub-inhibitory antibiotic treatments.* ISME Communications, 2022. **2**(1): p. 1-7.
18. Barrick, J.E. and R.E. Lenski, *Genome dynamics during experimental evolution.* Nature Reviews Genetics, 2013. **14**(12): p. 827-839.

19. Jagdish, T. and A.N.N. Ba, *Microbial experimental evolution in a massively multiplexed and high-throughput era.* Current Opinion in Genetics & Development, 2022. **75**: p. 101943.
20. Mee, J.A. and S. Yeaman, *Unpacking conditional neutrality: genomic signatures of selection on conditionally beneficial and conditionally deleterious mutations.* The American Naturalist, 2019. **194**(4): p. 529-540.
21. Manriquez, B., D. Muller, and C. Prigent-Combaret, *Experimental evolution in plant-microbe systems: A tool for deciphering the functioning and evolution of plant-associated microbial communities.* Frontiers in Microbiology, 2021. **12**: p. 896.
22. McDonald, M.J., *Microbial experimental evolution–a proving ground for evolutionary theory and a tool for discovery.* EMBO reports, 2019. **20**(8): p. e46992.
23. Van den Bergh, B., et al., *Experimental design, population dynamics, and diversity in microbial experimental evolution.* Microbiology and Molecular Biology Reviews, 2018. **82**(3): p. e00008-18.
24. Baym, M., et al., *Spatiotemporal microbial evolution on antibiotic landscapes.* Science, 2016. **353**(6304): p. 1147-1151.
25. Rainey, P.B. and M. Travisano, *Adaptive radiation in a heterogeneous environment.* Nature, 1998. **394**(6688): p. 69-72.
26. Baveye, P.C. and M. Laba, *Moving away from the geostatistical lamppost: Why, where, and how does the spatial heterogeneity of soils matter?* Ecological Modelling, 2015. **298**: p. 24-38.
27. Wilpiszeski, R.L., et al., *Soil aggregate microbial communities: towards understanding microbiome interactions at biologically relevant scales.* Applied and environmental microbiology, 2019. **85**(14): p. e00324-19.
28. Bach, E.M., et al., *Greatest soil microbial diversity found in micro-habitats.* Soil Biology and Biochemistry, 2018. **118**: p. 217-226.
29. Lenski, R.E., *Experimental evolution and the dynamics of adaptation and genome evolution in microbial populations.* The ISME Journal, 2017. **11**(10): p. 2181-2194.
30. Sokol, N.W., et al., *Life and death in the soil microbiome: How ecological processes influence biogeochemistry.* Nature Reviews Microbiology, 2022: p. 1-16.
31. Caro, T.A., et al., *Hydrogen stable isotope probing of lipids demonstrates slow rates of microbial growth in soil.* Proceedings of the National Academy of Sciences, 2023. **120**(16): p. e2211625120.
32. Lennon, J.T. and S.E. Jones, *Microbial seed banks: the ecological and evolutionary implications of dormancy.* Nature reviews microbiology, 2011. **9**(2): p. 119.
33. Brockhurst, M.A., et al., *Niche occupation limits adaptive radiation in experimental microcosms.* PLoS One, 2007. **2**(2): p. e193.
34. Gómez, P. and A. Buckling, *Real-time microbial adaptive diversification in soil.* Ecology letters, 2013. **16**(5): p. 650-655.
35. Gómez, P. and A. Buckling, *Coevolution with phages does not influence the evolution of bacterial mutation rates in soil.* The ISME Journal, 2013. **7**(11): p. 2242-2244.
36. Gómez, P., et al., *Rapid decline of adaptation of Pseudomonas fluorescens to soil biotic environment.* Biology Letters, 2022. **18**(3): p. 20210593.
37. Padfield, D., et al., *Evolution of diversity explains the impact of pre-adaptation of a focal species on the structure of a natural microbial community.* The ISME journal, 2020. **14**(11): p. 2877-2889.

38. Maharjan, R.P., et al., *Simple Phenotypic Sweeps Hide Complex Genetic Changes in Populations.* Genome Biology and Evolution, 2015. **7**(2): p. 531-544.
39. Gupta, R.S., et al., *Robust demarcation of 17 distinct Bacillus species clades, proposed as novel Bacillaceae genera, by phylogenomics and comparative genomic analyses: description of Robertmurraya kyonggiensis sp. nov. and proposal for an emended genus Bacillus limiting it only to the members of the Subtilis and Cereus clades of species.* Int. J. System. Evol. Microbiol., 2020. **70**(11): p. 5753-5798.
40. Jiang, H., et al., *Role of halotolerant phosphate-solubilising bacteria on growth promotion of peanut (Arachis hypogaea) under saline soil.* Ann. Appl. Biol., 2019. **174**(1): p. 20-30.
41. Saeid, A., E. Prochownik, and J. Dobrowolska-Iwanek, *Phosphorus solubilization by Bacillus species.* Molecules, 2018. **23**(11): p. 2897.
42. Sharma, S.B., et al., *Phosphate solubilizing microbes: sustainable approach for managing phosphorus deficiency in agricultural soils.* SpringerPlus, 2013. **2**: p. 14.
43. McSpadden Gardener, B.B., *Ecology of Bacillus and Paenibacillus spp. in agricultural systems.* Phytopathology, 2004. **94**(11): p. 1252-1258.
44. Levinson, H.S. and M.T. Hyatt, *Sequence of events during Bacillus megaterium spore germination.* Journal of bacteriology, 1966. **91**(5): p. 1811-1818.
45. Takors, R., *Scale-up of microbial processes: impacts, tools and open questions.* Journal of biotechnology, 2012. **160**(1-2): p. 3-9.
46. Lees, K., et al., *Soil sterilisation methods for use in OECD 106: How effective are they?* Chemosphere, 2018. **209**: p. 61-67.
47. McNamara, N., et al., *Effects of acute gamma irradiation on chemical, physical and biological properties of soils.* Applied Soil Ecology, 2003. **24**(2): p. 117-132.
48. Cairns, J., et al., *Genomic evolution of bacterial populations under coselection by antibiotics and phage.* Molecular Ecology, 2017. **26**(7): p. 1848-1859.
49. Castledine, M., D. Padfield, and A. Buckling, *Experimental (co) evolution in a multi-species microbial community results in local maladaptation.* Ecology Letters, 2020. **23**(11): p. 1673-1681.
50. Trivedi, U.H., et al., *Quality control of next-generation sequencing data without a reference.* Frontiers in Genetics, 2014. **5**(111).
51. Bolger, A.M., M. Lohse, and B. Usadel, *Trimmomatic: a flexible trimmer for Illumina sequence data.* Bioinformatics, 2014. **30**(15): p. 2114-2120.
52. Bankevich, A., et al., *SPAdes: a new genome assembly algorithm and its applications to single-cell sequencing.* Journal of computational biology, 2012. **19**(5): p. 455-477.
53. Li, H. and R. Durbin, *Fast and accurate short read alignment with Burrows–Wheeler transform.* Bioinformatics, 2009. **25**(14): p. 1754-1760.
54. Li, H., et al., *The Sequence Alignment/Map format and SAMtools.* Bioinformatics, 2009. **25**(16): p. 2078-2079.
55. Gurevich, A., et al., *QUAST: quality assessment tool for genome assemblies.* Bioinformatics, 2013. **29**(8): p. 1072-1075.
56. Seemann, T., *Prokka: rapid prokaryotic genome annotation.* Bioinformatics, 2014. **30**(14): p. 2068-2069.
57. Shwed, P.S., et al., *Complete Genome Sequences of Priestia megaterium Type and Clinical Strains Feature Complex Plasmid Arrays.* Microbiology Resource Announcements, 2021. **10**(27): p. e00403-21.

58. Deatherage, D.E. and J.E. Barrick, *Identification of mutations in laboratory-evolved microbes from next-generation sequencing data using breseq*, in *Engineering and analyzing multicellular systems*. 2014, Springer. p. 165-188.
59. Metzgar, D., et al., *The microsatellites of Escherichia coli: rapidly evolving repetitive DNAs in a non-pathogenic prokaryote.* Molecular microbiology, 2001. **39**(1): p. 183-190.
60. Roux, S., et al., *Ecology and molecular targets of hypermutation in the global microbiome.* Nature Communications, 2021. **12**(1): p. 3076.
61. Eppinger, M., et al., *Genome Sequences of the Biotechnologically Important Bacillus megaterium Strains QM B1551 and DSM319.* Journal of Bacteriology, 2011. **193**(16): p. 4199-4213.
62. Keats, B.J.B. and S.L. Sherman, *Chapter 13 - Population Genetics*, in *Emery and Rimoin's Principles and Practice of Medical Genetics (Sixth Edition)*, D. Rimoin, R. Pyeritz, and B. Korf, Editors. 2013, Academic Press: Oxford. p. 1-12.
63. Kaczanowska, M. and M. Rydén-Aulin, *Ribosome biogenesis and the translation process in Escherichia coli.* Microbiol Mol Biol Rev, 2007. **71**(3): p. 477-94.
64. Lenski, R.E., *What is adaptation by natural selection? Perspectives of an experimental microbiologist.* PLoS genetics, 2017. **13**(4): p. e1006668.
65. Castilla-Llorente, V., et al., *Spo0A, the key transcriptional regulator for entrance into sporulation, is an inhibitor of DNA replication.* Embo j, 2006. **25**(16): p. 3890-9.
66. Jiang, M., et al., *Multiple histidine kinases regulate entry into stationary phase and sporulation in Bacillus subtilis.* Molecular microbiology, 2000. **38**(3): p. 535-542.
67. Varughese, K.I., et al., *Formation of a novel four-helix bundle and molecular recognition sites by dimerization of a response regulator phosphotransferase.* Molecular cell, 1998. **2**(4): p. 485-493.
68. Kvitek, D.J. and G. Sherlock, *Whole Genome, Whole Population Sequencing Reveals That Loss of Signaling Networks Is the Major Adaptive Strategy in a Constant Environment.* PLOS Genetics, 2013. **9**(11): p. e1003972.
69. Zhao, H., et al., *DNA complexed structure of the key transcription factor initiating development in sporulating bacteria.* Structure, 2002. **10**(8): p. 1041-1050.
70. Maughan, H., et al., *The Roles of Mutation Accumulation and Selection in Loss of Sporulation in Experimental Populations of Bacillus subtilis.* Genetics, 2007. **177**(2): p. 937-948.
71. Dragosits, M. and D. Mattanovich, *Adaptive laboratory evolution–principles and applications for biotechnology.* Microbial cell factories, 2013. **12**(1): p. 64.
72. Hottes, A.K., et al., *Bacterial Adaptation through Loss of Function.* PLOS Genetics, 2013. **9**(7): p. e1003617.
73. Berninger, T., et al., *Maintenance and assessment of cell viability in formulation of non-sporulating bacterial inoculants.* Microbial biotechnology, 2018. **11**(2): p. 277-301.
74. Hamon, M.A. and B.A. Lazazzera, *The sporulation transcription factor Spo0A is required for biofilm development in Bacillus subtilis.* Molecular microbiology, 2001. **42**(5): p. 1199-1209.
75. Bishop, A.H., P.A. Rachwal, and A. Vaid, *Identification of Genes Required by Bacillus thuringiensis for Survival in Soil by Transposon-Directed Insertion Site Sequencing.* Current Microbiology, 2014. **68**(4): p. 477-485.
76. Rinaudi, L.V. and W. Giordano, *An integrated view of biofilm formation in rhizobia.* FEMS Microbiology Letters, 2010. **304**(1): p. 1-11.

77. Vilas-Bôas, L.A., et al., *Survival and conjugation of Bacillus thuringiensis in a soil microcosm.* FEMS Microbiology Ecology, 2000. **31**(3): p. 255-259.
78. Zheng, X.Y. and J.B. Sinclair, *The effects of traits of Bacillus megaterium onseed and root colonization and their correlation withthe suppression of Rhizoctonia root rot of soybean.* BioControl, 2000. **45**(2): p. 223-243.
79. Hartz, P., et al., *Development and application of a highly efficient CRISPR-Cas9 system for genome engineering in Bacillus megaterium.* Journal of Biotechnology, 2021. **329**: p. 170-179.
80. Borer, B. and D. Or, *Bacterial age distribution in soil–Generational gaps in adjacent hot and cold spots.* PLoS computational biology, 2022. **18**(2): p. e1009857.
81. Bosshard, L., et al., *Dissection of the mutation accumulation process during bacterial range expansions.* BMC Genomics, 2020. **21**(1): p. 253.
82. Amandine, C., et al., *Unraveling coevolutionary dynamics using ecological genomics.* Trends in Genetics, 2022.
83. Bell, G. and A. Gonzalez, *Adaptation and evolutionary rescue in metapopulations experiencing environmental deterioration.* Science, 2011. **332**(6035): p. 1327-1330.
84. Hall, J.P.J., et al., *Positive selection inhibits gene mobilization and transfer in soil bacterial communities.* Nature Ecology & Evolution, 2017. **1**(9): p. 1348-1353.
85. Deng, L., et al., *Characterization of a Two-Component System Transcriptional Regulator, LtdR, That Impacts Group B Streptococcal Colonization and Disease.* Infection and Immunity, 2018. **86**(7): p. e00822-17.
86. Goosen, N. and G.F. Moolenaar, *Repair of UV damage in bacteria.* DNA Repair, 2008. **7**(3): p. 353-379.
87. Mosca, C., et al., *Over-Expression of UV-Damage DNA Repair Genes and Ribonucleic Acid Persistence Contribute to the Resilience of Dried Biofilms of the Desert Cyanobacetrium Chroococcidiopsis Exposed to Mars-Like UV Flux and Long-Term Desiccation.* Front Microbiol, 2019. **10**: p. 2312.
88. Wang, Q., et al., *Enhanced production of L-arginine by improving carbamoyl phosphate supply in metabolically engineered Corynebacterium crenatum.* Applied Microbiology and Biotechnology, 2021. **105**(8): p. 3265-3276.
89. Wicht, D.K., *The reduced flavin-dependent monooxygenase SfnG converts dimethylsulfone to methanesulfinate.* Arch Biochem Biophys, 2016. **604**: p. 159-66.
90. Shibata, S., et al., *FliK regulates flagellar hook length as an internal ruler.* Molecular microbiology, 2007. **64**(5): p. 1404-1415.
91. Ali, V., Y. Shigeta, and T. Nozaki, *Molecular and structural characterization of NADPH-dependent d-glycerate dehydrogenase from the enteric parasitic protist Entamoeba histolytica.* Biochem J, 2003. **375**(Pt 3): p. 729-36.
92. Klewing, A., et al., *Resistance to serine in Bacillus subtilis: identification of the serine transporter YbeC and of a metabolic network that links serine and threonine metabolism.* Environ Microbiol, 2020. **22**(9): p. 3937-3949.
93. Carles, L., et al., *Genomic analysis of the Bacillus megaterium Mes11: New insights into nitroreductase genes associated with the degradation of mesotrione.* International Biodeterioration & Biodegradation, 2021. **162**: p. 105254.
94. Deniziak, M. and J. Barciszewski, *Methionyl-tRNA synthetase.* Acta biochimica polonica, 2001. **48**(2): p. 337-350.

95. Köster, W., *ABC transporter-mediated uptake of iron, siderophores, heme and vitamin B12.* Research in microbiology, 2001. **152**(3-4): p. 291-301.
96. Costa, F.G. and J.C. Escalante-Semerena, *Localization and interaction studies of the Salmonella enterica ethanolamine ammonia-lyase (EutBC), its reactivase (EutA), and the EutT corrinoid adenosyltransferase.* Molecular Microbiology, 2022. **118**(3): p. 191-207.
97. He, T., et al., *Identification of bacteria with β-galactosidase activity in faeces from lactase non-persistent subjects.* FEMS Microbiology Ecology, 2005. **54**(3): p. 463-469.
98. Pidutti, P., et al., *Purification and characterization of ribosomal proteins L27 and L30 having antimicrobial activity produced by the Lactobacillus salivarius SGL 03.* Journal of applied microbiology, 2018. **124**(2): p. 398-407.
99. Hosie, A.H.F. and P.S. Poole, *Bacterial ABC transporters of amino acids.* Research in Microbiology, 2001. **152**(3): p. 259-270.
100. Kaplan, E., et al., *Insights into bacterial lipoprotein trafficking from a structure of LolA bound to the LolC periplasmic domain.* Proceedings of the National Academy of Sciences, 2018. **115**(31): p. E7389-E7397.
101. Luthra, A., et al., *Structure and mechanism of a bacterial t6A biosynthesis system.* Nucleic Acids Research, 2018. **46**(3): p. 1395-1411.
102. Missoury, S., et al., *The structure of the TsaB/TsaD/TsaE complex reveals an unexpected mechanism for the bacterial t6A tRNA-modification.* Nucleic Acids Research, 2018. **46**(11): p. 5850-5860.
103. Kadaba, N.S., et al., *The High-Affinity <i>E. coli</i> Methionine ABC Transporter: Structure and Allosteric Regulation.* Science, 2008. **321**(5886): p. 250-253.
104. Boquet, P., *Small GTP binding proteins and bacterial virulence.* Microbes and Infection, 2000. **2**(7): p. 837-843.
105. Lambrecht, J.A. and D.M. Downs, *Anthranilate Phosphoribosyl Transferase (TrpD) Generates Phosphoribosylamine for Thiamine Synthesis from Enamines and Phosphoribosyl Pyrophosphate.* ACS Chemical Biology, 2013. **8**(1): p. 242-248.
106. Agarwal, G., R.D. Gitaitis, and B. Dutta, *Pan-Genome of Novel Pantoea stewartii subsp. indologenes Reveals Genes Involved in Onion Pathogenicity and Evidence of Lateral Gene Transfer.* Microorganisms, 2021. **9**(8): p. 1761.
107. Wendt, K.U., A. Lenhart, and G.E. Schulz, *The structure of the membrane protein squalene-hopene cyclase at 2.0 å resolution 11Edited by D. C. Rees.* Journal of Molecular Biology, 1999. **286**(1): p. 175-187.
108. Munro, A.W., et al., *P450 BM3: the very model of a modern flavocytochrome.* Trends in Biochemical Sciences, 2002. **27**(5): p. 250-257.
109. Setlow, B., D. Sun, and P. Setlow, *Interaction between DNA and alpha/beta-type small, acid-soluble spore proteins: a new class of DNA-binding protein.* Journal of bacteriology, 1992. **174**(7): p. 2312-2322.
110. Matange, N., M. Podobnik, and S.S. Visweswariah, *Metallophosphoesterases: structural fidelity with functional promiscuity.* Biochem J, 2015. **467**(2): p. 201-16.
111. Luthra, A., et al., *Conformational communication mediates the reset step in t6A biosynthesis.* Nucleic Acids Research, 2019. **47**(12): p. 6551-6567.
112. Jin, F., Y. Huang, and M. Hattori, *Recent Advances in the Structural Biology of Mg2+ Channels and Transporters.* Journal of Molecular Biology, 2022. **434**(19): p. 167729.
113. Hefti, M.H., et al., *The PAS fold: A redefinition of the PAS domain based upon structural prediction.* European Journal of Biochemistry, 2004. **271**(6): p. 1198-1208.

114. Zhang, H., et al., *Insights into the Catalytic Mechanism of 16S rRNA Methyltransferase RsmE (m3U1498) from Crystal and Solution Structures.* Journal of Molecular Biology, 2012. **423**(4): p. 576-589.

115. Blatch, G.L. and M. Lässle, *The tetratricopeptide repeat: a structural motif mediating protein-protein interactions.* Bioessays, 1999. **21**(11): p. 932-939.

116. Doerrler, W.T., et al., *New Functions for the Ancient DedA Membrane Protein Family.* Journal of Bacteriology, 2013. **195**(1): p. 3-11.
